# Germline MBD4 deficiency causes a multi-tumor predisposition syndrome

**DOI:** 10.1016/j.ajhg.2022.03.018

**Published:** 2022-04-22

**Authors:** Claire Palles, Hannah D. West, Edward Chew, Sara Galavotti, Christoffer Flensburg, Judith E. Grolleman, Erik A.M. Jansen, Helen Curley, Laura Chegwidden, Edward H. Arbe-Barnes, Nicola Lander, Rebekah Truscott, Judith Pagan, Ashish Bajel, Kitty Sherwood, Lynn Martin, Huw Thomas, Demetra Georgiou, Florentia Fostira, Yael Goldberg, David J. Adams, Simone A.M. van der Biezen, Michael Christie, Mark Clendenning, Laura E. Thomas, Constantinos Deltas, Aleksandar J. Dimovski, Dagmara Dymerska, Jan Lubinski, Khalid Mahmood, Rachel S. van der Post, Mathijs Sanders, Jürgen Weitz, Jenny C. Taylor, Clare Turnbull, Lilian Vreede, Tom van Wezel, Celina Whalley, Claudia Arnedo-Pac, Giulio Caravagna, William Cross, Daniel Chubb, Anna Frangou, Andreas J. Gruber, Ben Kinnersley, Boris Noyvert, David Church, Trevor Graham, Richard Houlston, Nuria Lopez-Bigas, Andrea Sottoriva, David Wedge, Genomics England Research Consortium, The CORGI Consortium, WGS500 Consortium, Mark A. Jenkins, Roland P. Kuiper, Andrew W. Roberts, Jeremy P. Cheadle, Marjolijn J.L. Ligtenberg, Nicoline Hoogerbrugge, Viktor H. Koelzer, Andres Dacal Rivas, Ingrid M. Winship, Clara Ruiz Ponte, Daniel D. Buchanan, Derek G. Power, Andrew Green, Ian P.M. Tomlinson, Julian R. Sampson, Ian J. Majewski, Richarda M. de Voer

**Affiliations:** 1 Institute of Cancer and Genomic Sciences, College of Medical and Dental Science, University of Birmingham, Edgbaston, Birmingham B15 2TT, UK; 2 Institute of Medical Genetics, Division of Cancer and Genetics, Cardiff University, School of Medicine, Cardiff, UK; 3 Walter and Eliza Hall Institute of Medical Research, Parkville, VIC 3052, Australia; 4 Department of Human Genetics, Radboud Institute for Molecular Life Sciences, Radboud University Medical Center, 6525 Nijmegen, the Netherlands; 5 Wellcome Trust Centre for Human Genetics, University of Oxford, Oxford OX3 7BN, UK; 6 Molecular Genetics Laboratory, South East Scotland Genetic Service, Western General Hospital, Crewe Road, Edinburgh EH4 2XU, UK; 7 Peter MacCallum Cancer Center and Royal Melbourne Hospital, Victorian Comprehensive Cancer Centre, Parkville, VIC, Australia; 8 Edinburgh Cancer Research Centre, IGMM, University of Edinburgh, Crewe Road, Edinburgh EH4 2XR, UK; 9 St Mark’s Hospital, Imperial College London, London, UK; 10 Genomic Medicine, Imperial College Healthcare Trust and North West Thames Regional Genetics Service, Northwick Park, Harrow, UK; 11 Molecular Diagnostics Laboratory, NCSR Demokritos, Athens, Greece; 12 Raphael Recanati Genetic Institute, Rabin Medical Center – Beilinson Hospital, Petach Tikva, Israel; 13 Sackler Faculty of Medicine, Tel Aviv University, Tel Aviv, Israel; 14 Wellcome Trust Sanger Institute, Wellcome Genome Campus, Hinxton, Cambridge CB10 1SA, UK; 15 Colorectal Oncogenomics Group, Department of Clinical Pathology, Melbourne Medical School, The University of Melbourne, Parkville, VIC, Australia; 16 University of Melbourne Centre for Cancer Research, Victorian Comprehensive Cancer Centre, Parkville, VIC, Australia; 17 Institute of Life Sciences, Swansea University, Swansea SA28PP, UK; 18 Center of Excellence in Biobanking and Biomedical Research and Molecular Medicine Research Center, University of Cyprus Medical School, Nicosia, Cyprus; 19 Center for Biomolecular Pharmaceutical Analyzes, UKIM Faculty of Pharmacy, 1000 Skopje, Republic of Macedonia; 20 Hereditary Cancer Center, Department of Genetics and Pathology, Pomeranian Medical University, 70-111 Szczecin, Poland; 21 Department of Pathology, Radboud Institute for Molecular Life Sciences, Radboud University Medical Center, 6525 Nijmegen, the Netherlands; 22 Department of Hematology, Erasmus University Medical Center, Rotterdam, the Netherlands; 23 Department of Surgical Research, Universitätsklinikum Carl Gustav Carus, Technische Universität Dresden, 01307 Dresden, Germany; 24 Oxford NIHR Biomedical Research Centre, Wellcome Trust Centre for Human Genetics, University of Oxford, Oxford OX3 7BN, UK; 25 Institute of Cancer Research, Cotswold Road, Sutton, Surrey SM2 5NG, UK; 26 Department of Pathology, Leiden University Medical Center, 2300 Leiden, the Netherlands; 27 Institute for Research in Biomedicine, The Barcelona Institute of Science and Technology, Barcelona, Spain; 28 Cancer Institute, University College London, 72 Huntley Street, London WC1E 6BT, UK; 29 Manchester Interdisciplinary Biocentre, University of Manchester, Manchester M1 7DN, UK; 30 Barts Cancer Institute, Barts and The London School of Medicine and Dentistry, Queen Mary University of London, London, UK; 31 Centre for Epidemiology and Biostatistics, Melbourne School of Population and Global Health, The University of Melbourne, Parkville, VIC, Australia; 32 Princess Máxima Center for Pediatric Oncology, 3584 Utrecht, the Netherlands; 33 University of Melbourne, Department of Medical Biology, 1G Royal Parade, Parkville, VIC 3052, Australia; 34 Department of Pathology and Molecular Pathology, University Hospital Zurich, University of Zurich, Zürich, Switzerland; 35 Servicio de Digestivo, Hospital Lucus Augusti, Instituto de Investigación Sanitaria de Santiago, Lugo, Galicia, Spain; 36 Genomic Medicine and Family Cancer Clinic, Royal Melbourne Hospital, Melbourne, VIC, Australia; 37 Department of Medicine, Melbourne Medical School, Faculty of Medicine, Dentistry and Health Sciences, University of Melbourne, Melbourne, VIC, Australia; 38 Fundación Pública Galega de Medicina Xenómica SERGAS, Grupo de Medicina Xenómica-USC, Instituto de Investigación Sanitaria de Santiago, Centro de Investigación Biomédica en Red de Enfermedades Raras, Santiago de Compostela, Galicia, Spain; 39 Department of Medical Oncology, Cork University Hospital, Cork, Ireland; 40 Department of Clinical Genetics, Children’s Health Ireland, Dublin, Ireland; School of Medicine University College, Dublin, Ireland

**Keywords:** polyposis, colorectal cancer, 5′-methylcytosine deamination, mutational signature, mutator phenotype

## Abstract

We report an autosomal recessive, multi-organ tumor predisposition syndrome, caused by bi-allelic loss-of-function germline variants in the base excision repair (BER) gene *MBD4*. We identified five individuals with bi-allelic *MBD4* variants within four families and these individuals had a personal and/or family history of adenomatous colorectal polyposis, acute myeloid leukemia, and uveal melanoma. *MBD4* encodes a glycosylase involved in repair of G:T mismatches resulting from deamination of 5′-methylcytosine. The colorectal adenomas from MBD4-deficient individuals showed a mutator phenotype attributable to mutational signature SBS1, consistent with the function of MBD4. MBD4-deficient polyps harbored somatic mutations in similar driver genes to sporadic colorectal tumors, although *AMER1* mutations were more common and *KRAS* mutations less frequent. Our findings expand the role of BER deficiencies in tumor predisposition. Inclusion of *MBD4* in genetic testing for polyposis and multi-tumor phenotypes is warranted to improve disease management.

## Main text

Inherited defects in DNA repair are responsible for a group of genetic tumor risk syndromes that are characterized by adenomatous polyposis, colorectal cancer (CRC), and extracolonic neoplasms. These syndromes include dominantly inherited polymerase proofreading-associated polyposis (PPAP) caused by pathogenic variants in the polymerase proofreading domains of *POLE* (MIM: 615083) and *POLD1* (MIM: 612591)^1^ and recessively inherited conditions caused by variants in genes involved in mismatch repair (*PMS2* [MIM: 614337], *MSH6* [MIM: 600678], *MSH2* [MIM: 120435], *MLH1* [MIM: 609310])^2^^,^^3^ and base-excision repair (BER) (*MUTYH* [MIM: 608456] and *NTHL1* [MIM: 616415]).^4^^,^^5^ Mechanistically, defective DNA repair appears to lead to an increase in the somatic mutation rate and accumulation of somatic mutations in cancer driver genes such as *APC* (MIM: 611731), *KRAS* (MIM: 190070), and *TP53* (MIM: 191170). Previous studies have linked the specific defects in DNA repair genes to mutational signatures.^6^, ^7^, ^8^, ^9^

Genetic testing currently fails to identify a cause in a significant proportion of individuals who develop multiple colorectal adenomas. It is important to identify any remaining polyposis genes in order to plan appropriate tumor surveillance for affected individuals and their relatives. Here, by applying whole-genome and whole-exome sequencing (WGS and WES), we identified loss-of-protein-function (LOF) variants of the BER gene *MBD4* as the cause of an autosomal recessive syndrome of colorectal polyposis and extracolonic neoplasia.

We performed WGS or WES of constitutional DNA in a cohort of 309 individuals, from 198 apparently unrelated families, who were affected by multiple colorectal adenomas or familial CRC. For all individuals included in our study, routine diagnostic molecular genetic testing failed to detect pathogenic germline variants in known CRC and polyposis predisposing genes (detailed cohort descriptions in supplemental methods). The study received ethical approval from UK NHS Research Ethics Committees (REC numbers 06/Q1702/99 and 12/WA/0071), the Human Research Ethics Committees at the University of Melbourne (HREC #1954921), and the Radboudumc CMO Local Ethics Committee (#2015/2172). All participants provided written informed consent. Following WGS or WES, we prioritized the identification of coding germline variants predicted to cause LOF. This approach identified two individuals with bi-allelic frameshift variants in *MBD4*. *MBD4* encodes a BER glycosylase that repairs G:T mismatches resulting from the deamination of 5′-methylcytosine (5mC). Simplex individual D:II-1 was homozygous for a 4-bp *MBD4* deletion (GenBank: NM_003925.2: c.612_615del [p.Ser205Thrfs^∗^9]; Figures S1A and S1E–S1H) and the other (CRDFF-292:II-3) was homozygous for an adenine duplication (GenBank: NM_003925.2: c.939dup [p.Glu314Argfs^∗^13]; Figure S1B). Region of homozygosity analysis did not suggest consanguinity in either of the two individuals (data not shown). Both variants were exceedingly rare in gnomAD (allele frequencies 0.0000399 and 0.000653, respectively), although the c.939dup variant is one of the most common LOF variants in *MBD4* in gnomAD and was found across multiple populations. No individuals with homozygous germline LOF *MBD4* variants were found in gnomAD, the UK 100,000 Genome Project (100KGP), or the whole-genome-sequenced individuals in UK Biobank.

Next, we undertook targeted sequencing of *MBD4* in replication cohorts comprising a total of 1,611 individuals with at least ten colorectal adenomas, familial or early onset CRC, or CRC in combination with other tumors. This identified one additional, unrelated individual (CRDFF-336-1:II-1) who was homozygous for the same adenine duplication (GenBank: NM_003925.2: c.939dup; Figure S1C) and four heterozygous carriers of LOF variants in *MBD4*. While the frequency of heterozygous carriers was significantly higher than in gnomAD (4/1,611 versus 48/64,600; p = 0.0381, Fisher’s exact), we did not confirm this enrichment in the 100KGP and UK Biobank datasets (all comparisons p > 0.05, Fisher’s exact; Table S3).

**Figure 1:**
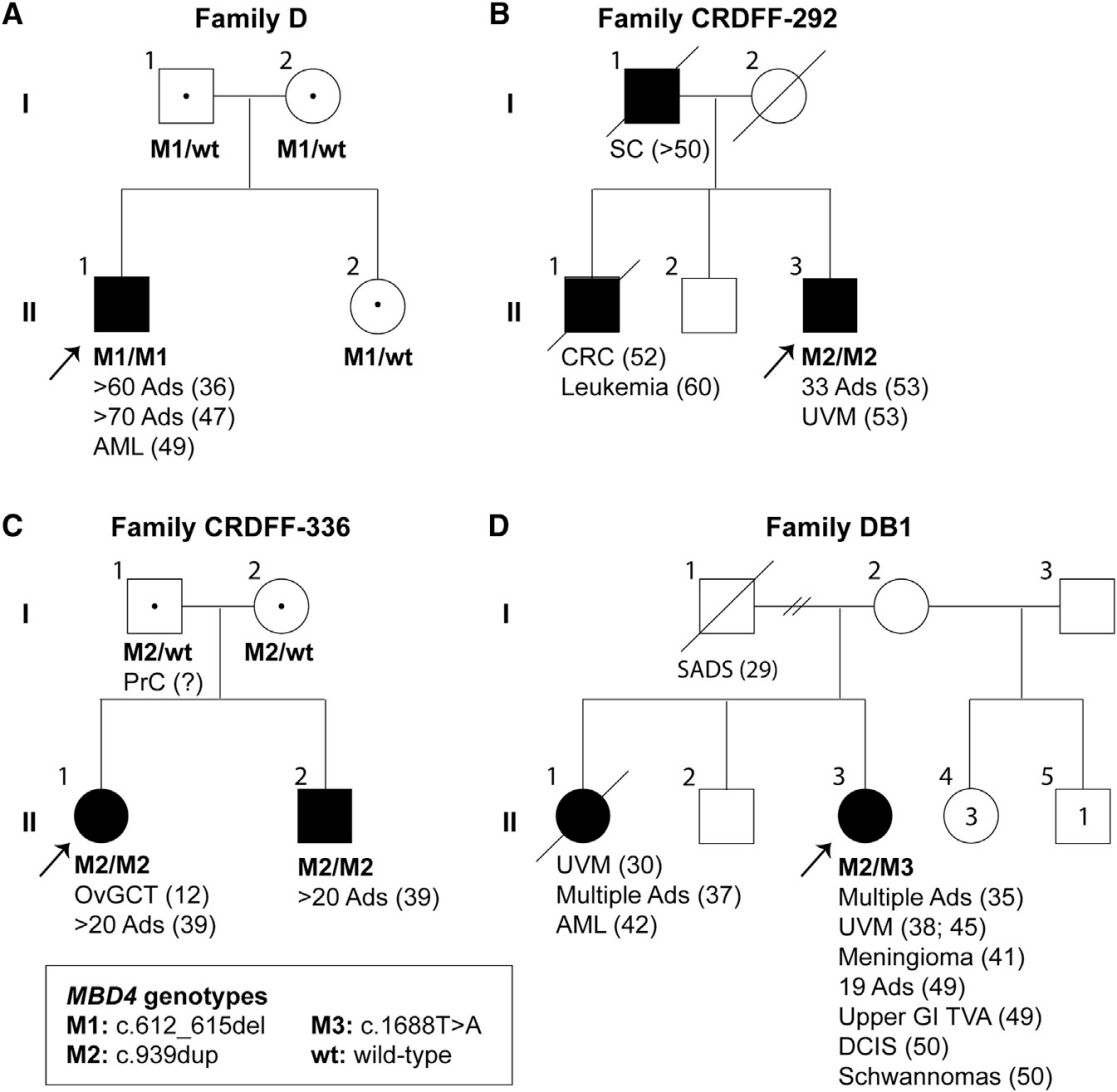
Pedigrees of individuals with MBD4 deficiency (A) Pedigree of simplex individual D:II-1 with a homozygous *MBD4* loss-of-function (c.612_615del) variant. For colorectal adenomas, we show the cumulative tumor numbers from age at first presentation and screening colonoscopy to age at last contact (see also Figures S1A and S1E–S1H). (B) Pedigree of Family CRDFF-292 with a homozygous *MBD4* loss-of-function (c.939dup) variant. For colorectal adenomas, we show the total tumor numbers identified from panproctocolectomy at age 53 (see also Figure S1B). (C) Pedigree of Family CRDFF-336 with a homozygous *MBD4* loss-of-function (c.939dup) variant. For colorectal adenomas, we show the total tumor numbers identified from panproctocolectomy at 39 years of age (see also Figure S1C). (D) Pedigree of Family DB1 with the bi-allelic *MBD4* loss-of-function c.939dup and c.1688T>A variants (see also Figure S1D). Abbreviations: Ads, colorectal adenomas; AML, acute myeloid leukemia; CRC, colorectal cancer; DCIS, ductal carcinoma *in situ* of the breast; OvGCT, ovarian granulosa cell tumor; PrC, prostate cancer; SADS, sudden arrhythmia death syndrome; SC, stomach cancer; UVM, uveal melanoma; upper GI TVA, upper gastrointestinal ampullary tubulovillus adenoma; question mark, age unknown; MT, mutation; WT, wild-type. Arrows indicate index individuals. Number between brackets indicate age at diagnosis.

The pedigrees of the three individuals with homozygous *MBD4* germline variants are shown in Figure 1. After genotyping of available additional family members, all were compatible with an autosomal recessive trait. Most individuals with bi-allelic LOF variants in *MBD4* developed multiple colorectal adenomas and an extracolonic neoplasm (Table 1). Simplex individual D:II-1 (Figure 1A) was found to have approximately 60 colorectal adenomas at initial colonoscopy at 36 years of age and at least 70 adenomas were identified at panproctocolectomy at 47 years of age (Figure S1H). 7 months after surgery, he was diagnosed with myelodysplastic syndrome (MDS) that progressed to acute myeloid leukemia (AML) within 3 months. CRDFF-292-1 (individual II-3 in Figure 1B) had 33 colorectal adenomas at panproctocolectomy at 53 years of age and was diagnosed concurrently with a uveal melanoma. A CT scan also revealed multiple liver cysts and multiple, bilateral small renal cysts. His brother (individual II-1 in Figure 1B) had been diagnosed with colorectal cancer at 52 years of age and had died of leukemia aged 60, but no material was available for genetic testing. CRDFF-336-1 (individual II-1 in Figure 1C) had 20 colorectal adenomas at panproctocolectomy at 39 years of age and previously had surgical removal of an ovarian granulosa cell tumor at 12 years of age. Genotyping confirmed that her brother (CRDFF-336-2, individual II-2 in Figure 1C) was also homozygous for the adenine duplication (c.939dup), and colonoscopy at 39 years of age revealed approximately 20 colorectal polyps that were confirmed histologically to be adenomas with low grade dysplasia.

**Table 1:** Clinical phenotype of individuals with bi-allelic germline *MBD4* loss-of-protein-function variants

**Individual**	**cDNA change (GenBank: NM_003925.2)**	**Amino acid change**	**M/F**	**Malignancies**	**Polyps**	**Benign lesions**
D:II-1	c.612_615del (homozygous)	p.Ser205Thrfs^∗^9	M	AML (49)	>130 A	N/A
CRDFF-292-1:II-3	c.939dup (homozygous)	p.Glu314Argfs^∗^13	M	UVM (53)	33 A	liver cysts (53), bilateral small renal cysts (53)
CRDFF-336-1:II-1	c.939dup (homozygous)	p.Glu314Argfs^∗^13	F	OvGCT (12)	>20 A	N/A
CRDFF-336-2:II-2	c.939dup (homozygous)	p.Glu314Argfs^∗^13	M	N/A	>20 A	N/A
DB1-70:II-3	c.939dup/c.1688T>A	p.Glu314Argfs^∗^13/p.Leu563^∗^	F	UVM (38, 45)	multiple A (35); 19 A (49); upper GI TVA (49)	meningioma (41); DCIS (50); schwannomas (50)
WEHI-2^10^	c.939dup/c.1562−1G>T	p.Glu314Argfs^∗^13/abnormal splicing	F	AML (34); CRC (40)	17 A	N/A
WEHI-AML-1^10^	c.939dup/c.1562−1G>T	p.Glu314Argfs^∗^13/abnormal splicing	F	AML (31)	no colonoscopy performed	N/A
EMC-AML-1^10^	c.1699_1701del (homozygous)	p.His567del	M	AML (33)	multiple A	N/A

M, male; F, female; AML, acute myeloid leukemia; UVM, uveal melanoma; OvGCT, ovarian granulosa cell tumor; CRC, colorectal cancer; A, colorectal adenomas (numbers indicate total cumulative number of colorectal polyps unless stated otherwise); upper GI TVA, upper gastrointestinal tract tubulovillus adenoma; DCIS, ductal carcinoma *in situ* of the breast; N/A not applicable. Numbers in parentheses refer to the age of diagnosis of the affected individual. Unspecified number of polyps is indicated as “multiple.”

Two of three individuals with AML previously identified to have MBD4 deficiency were noted to have colorectal polyps, without information on their type or multiplicity.^10^ We therefore obtained more comprehensive clinicopathological information on their colorectal tumors (Table 1). Individual WEHI-2 (previously WEHI-AML-2^10^) developed a total of 17 colorectal polyps over a period of 22 years from the age of 18 years. Histological assessment classified all available polyps (n = 12) as tubular adenomas with mild-to-moderate dysplasia, and the majority (n = 7) were found in the rectum (Figure S1I). A moderately differentiated adenocarcinoma was found in the ascending colon at age 40 and the individual underwent a right hemicolectomy. Individual EMC-AML-1 developed multiple colonic polyps and underwent a hemicolectomy at age 31, although no polyp counts were reported and tissue blocks were unavailable for histological re-assessment. The third individual (WEHI-AML-1^10^) did not have gastrointestinal assessment prior to her death.

**Figure 2:**
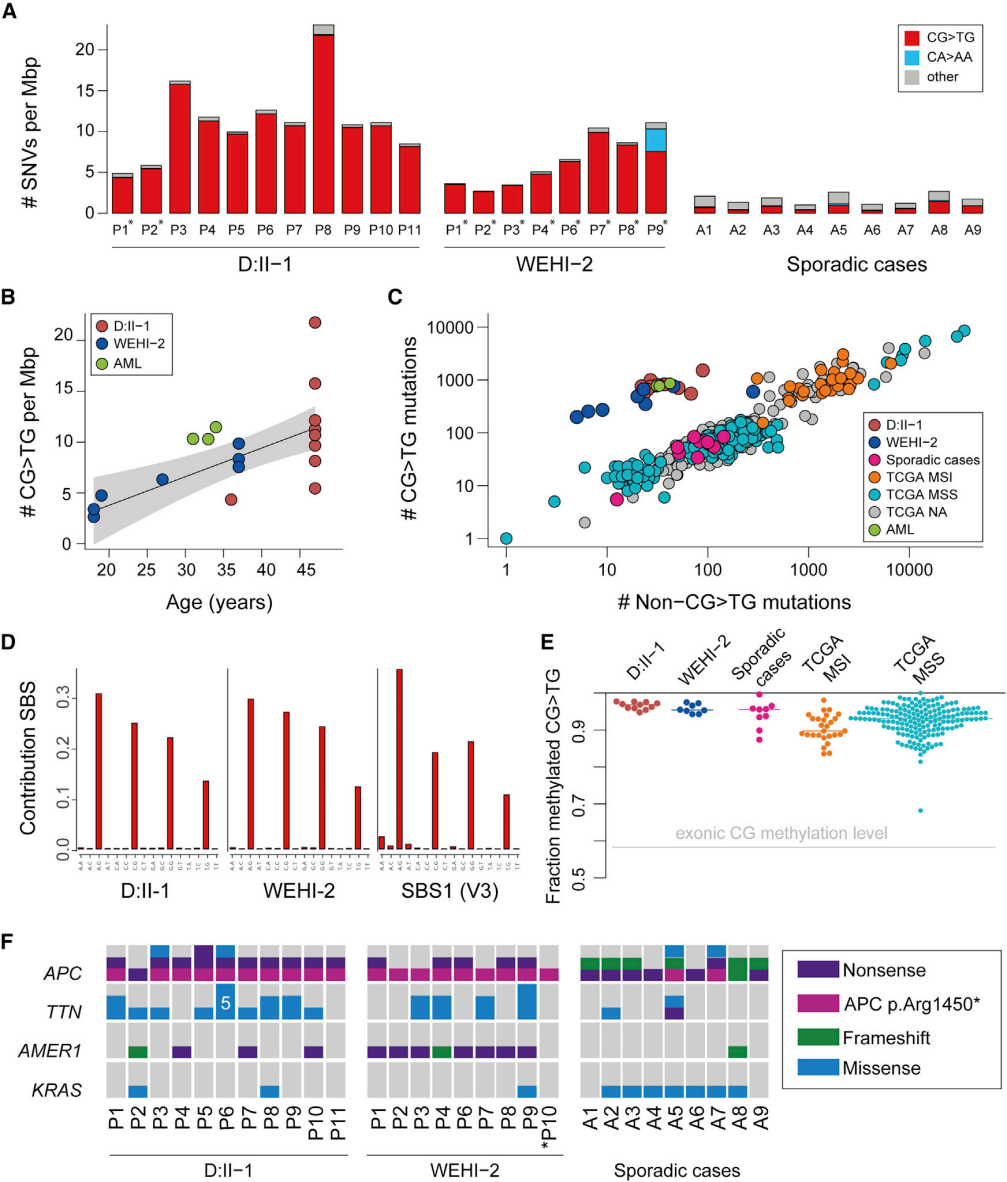
Somatic mutation burden and analysis of polyps of individuals with MBD4 deficiency (A) Somatic mutation rate for each polyp, formalin-fixed and paraffin-embedded samples indicated with asterisks (^∗^). The color of the bars represents mutations in different sequence contexts; red shows CG>TG mutations, blue shows CA>AA mutations (primarily detected in WEHI-2 P9), and gray represents other base contexts. The median value is presented for samples that had multi-region sequencing. Median mutation burden/Mb in fresh frozen adenomas of D:II-1 was 11.1 [range 8.5–23.3] compared to 1.8 in a set of nine fresh frozen sporadic adenomas [range 1.0–3.1] (see also Figures S1H–S1I for representative HE slides). (B) The number of somatic CG>TG mutations detected in WES data is plotted as function of age. The linear fit is shown, together with 95% confidence intervals (gray shading). (C) We assessed the contribution of deamination of 5mC to MBD4-deficient samples by comparing the number of CG>TG mutations to all other single-nucleotide mutations. The plot compares MBD4-deficient polyps and AMLs^12^ to sporadic polyps, and to colon and rectal cancers from The Cancer Genome Atlas (TCGA). MSI, microsatellite instability; MSI-H, MSI-high; MSS, microsatellite stable (“MSS” includes both MSS and MSI-low samples); TCGA NA, no MSI data available. (D) Extracted *de novo* signature SBS1^MBD4^ C>T panel from all polyps from D:II-1(left) and polyps P1–P8 of WEHI-2 (middle) and the C>T panel from COSMIC SBS1-v3 (right). (E) Fraction of mutated CpG sites that are methylated in normal sigmoid colon (beta value > 0.5 in WGBS data from the Roadmap Epigenomics Consortium^11^). Each point summarizes WES results from a sample and includes all sites with sufficient coverage in WGBS (n *=* 177–1,507 CG>TG mutations) and the median value is shown with a horizontal line. The gray line shows the fraction of methylated CG sites across all exons. (F) Oncoprint of driver gene mutation analysis of genes significantly different mutated compared to sporadic adenomas. For each polyp, the number and type of somatic mutation is shown. ^∗^Polyp P10 from WEHI-2 was sequenced with a targeted panel (see also Table S5).

We performed WES on DNA extracted from fresh-frozen or formalin-fixed paraffin-embedded (FFPE) tissue from 11 colorectal adenomas from D:II-1 and eight colorectal adenomas from WEHI-2 (Table S2). The mutation burden was increased significantly in colorectal adenomas from both individuals with MBD4 deficiency compared to previously published multi-region WES data from nine sporadic fresh-frozen adenomas^11^ (Figure 2A; Table S2). The excess mutations were almost all CpG>TpG transitions (>95%) that accumulated steadily over time (Figure 2B) and were significantly more prevalent (Fisher’s exact, p = 2.9 × 10^−7^) than in the sporadic colorectal adenomas (Figure 2C). The mutation spectrum was almost exclusively attributable to COSMIC mutational signature SBS1^12^ in colorectal adenomas from individuals with MBD4 deficiency, in contrast to sporadic colorectal adenomas (Figure 2D; Figures S2 and S3). This is fully consistent with a failure to repair G:T mismatches resulting from deamination of 5′-methylcytosine caused by loss of MBD4 function. Furthermore, virtually all mutated sites were methylated in normal colon (>96% of sites mutated compared to 58% of all exonic CpG sites; Figure 2E).

The driver genes mutated in MBD4-deficient adenomas were similar to those in sporadic adenomas and CRCs (Table S4). All MBD4-deficient adenomas (those that underwent WES and one additional adenoma from WEHI-2 that was targeted sequenced; see also Table S2 and the supplemental methods) harbored somatic driver mutations in *APC* with a significant enrichment of the CpG>TpG transition (GenBank: NM_000038.4: c.4348C>T) resulting in p.Arg1450^∗^, compared with sporadic adenomas and CRCs (Fisher’s exact; p < 0.00001; Figure 2F). MBD4-deficient adenomas harbored fewer *KRAS* mutations (three of 19 adenomas) than sporadic tumors (Fisher’s exact, p = 0.0028) but significantly more somatic mutations in *AMER1* (MIM: 300647) (12 of 19 adenomas; Fisher’s exact, p = 0.039) (Figure 2F). Overall, 88% of driver mutations in adenomas from individuals with MBD4 deficiency were CpG>TpG transitions compared to only 37% in sporadic adenomas (Table S4).

Incorporation of *MBD4* into diagnostic gene panels for colorectal polyposis, AML, and uveal melanoma at one of our centers has led to the identification of a further individual (DB1-70) with *MBD4*-associated neoplasia syndrome (MANS). DB1-70 (individual II-3 in Figure 1D) is compound heterozygous for the *MBD4* variants c.939dup and c.1688T>A (p.Leu563^∗^; Figure S1D). She developed multiple adenomatous polyps in the colon at age 35, underwent a left hemicolectomy at age 39, and had 19 adenomatous polyps removed from her residual colon at age 49. DB1-70 was also diagnosed with two uveal melanomas, one at age 38 and one at age 45 years, a meningioma at age 41, a ductal carcinoma *in situ* of the breast at age 50, and a chest wall and cervical schwannoma at age 50. At age 52, she was diagnosed with liver metastases from the uveal melanoma (Figure 1D). Her sister, for whom no material was available for genetic testing, was diagnosed with a uveal melanoma at age 30 and duodenal polyps and multiple adenomatous polyps in the colon, for which a right hemicolectomy was performed, at age 37. She was diagnosed with AML at age 42 and died a year later.

Following the discovery that bi-allelic *MBD4* LOF variants predispose to AML,^10^ we here show that inherited MBD4 deficiency causes a wider neoplastic syndrome including adenomatous polyposis with a colorectal phenotype similar to attenuated familial adenomatous polyposis (MIM: 175100) and to individuals with germline pathogenic variants in *MUTYH*, *NTHL1*, *POLE*, and *POLD1*. Loss of MBD4 function leads to an accumulation of somatic CpG>TpG mutations, including in well-known CRC driver genes, arising from spontaneous deamination of 5’-methylcytosine, creating a mutational signature very similar to COSMIC SBS1. We suggest the name *MBD4*-associated neoplasia syndrome (MANS) for this condition.

To date, colorectal polyposis, MDS/AML, and uveal melanoma appear to be the most common clinical manifestations of MANS. Thus far, to our knowledge, all individuals that have had a colonoscopy have had multiple colorectal polyps early in life, and most have experienced MDS/AML. Identification of individuals with bi-allelic *MBD4* pathogenic variants may inform their clinical management and that of their families. The identification and follow up of additional individuals will help to define the magnitude of cancer risks in MANS. In the interim, we propose colonoscopies every 2 years from age 18–20, or the date of diagnosis, a regimen often used for other BER-related polyposis syndromes.^13^^,^^14^ At least one of the AMLs in our study developed from MDS, and we have observed clonal hematopoiesis in others.^10^ We suggest regular follow up full blood counts for individuals with MANS if their initial presentation is with adenomatous polyposis. If the individual presents with AML, then we suggest genetic testing for any family member being considered as a haematopoietic stem cell donor, in keeping with current expert recommendations for managing inherited predisposition to myeloid malignancy.^15^^,^^16^ Given that heterozygous LOF *MBD4* variant carriers appear to be susceptible to uveal melanoma^17^, ^18^, ^19^ and our identification of uveal melanoma in three of eight individuals with MANS suggests annual ophthalmological surveillance may also be appropriate.^20^ The occurrence of a rare juvenile ovarian granulosa cell tumor in one of four females and schwannomas in another individual reported here is noteworthy and the spectrum of MBD4-deficiency-associated cancers may widen as further individuals with MANS are identified. In contrast to findings with uveal melanoma where heterozygotes for *MBD4* LOF variants appear to be at a 4- to 20-fold increased risk, our limited data show no convincing evidence for a comparable effect on the relative risk of developing polyposis and/or CRC. We cannot rule out the possibility that individuals heterozygous for an *MBD4* LOF variant have a small increased risk of CRC and/or polyposis,^21^ but at present, no colonoscopy surveillance beyond population screening or local guidelines based on familial history for CRC is recommended. Additionally, although it has been suggested that variable expression of MBD4 contributes to differences in DNA repair capacity,^22^ further investigation is required to determine whether this contributes to modify disease risk.

In conclusion, constitutional deficiency of MBD4 causes a rare genetic syndrome, MANS, that is characterized by the development of adenomatous polyposis and predisposition to AML. MBD4 deficiency results in an elevated mutation burden with a mutation spectrum very similar to COSMIC mutational signature SBS1. A high mutational burden is associated with a good prognosis in CRC, and we speculate that MANS CRCs may respond to immune checkpoint inhibitors, as has been reported in MBD4-deficient uveal melanomas.^17^^,^^18^ It is possible that such a strategy could also be used to treat other neoplasia in MANS. In the short term, genetic testing for MANS could be implemented readily by incorporating *MBD4* into existing gene panels used in diagnostic testing for adenomatous polyposis, CRC, early-onset AML, and uveal melanoma.

## Consortia

The members of Genomics England Research Consortium are John Ambrose, Prabhu Arumugam, Marta Bleda, Freya Boardman-Pretty, Christopher Boustred, Helen Brittain, Mark Caulfield, Georgia Chan, Tom Fowler, Adam Giess, Angela Hamblin, Shirley Henderson, Tim Hubbard, Rob Jackson, Louise Jones, Dalia Kasperaviciute, Melis Kayikci, Athanasios Kousathanas, Lea Lahnstein, Sarah Leigh, Ivone Leong, Javier Lopez, Fiona Maleady-Crowe, Meriel McEntagart, Federico Minneci, Loukas Moutsianas, Michael Mueller, Nirupa Murugaesu, Anna Need, Peter O’Donovan, Chris Odhams, Christine Patch, Daniel Perez-Gil, Mariana Pereira, John Pullinger, Tahrima Rahim, Augusto Rendon, Tim Rogers, Kevin Savage, Kushmita Sawant, Richard Scott, Afshan Siddiq, Alexander Sieghart, Samuel Smith, Alona Sosinsky, Alexander Stuckey, Mélanie Tanguy, Ana Tavares, Ellen Thomas, Simon Thompson, Arianna Tucci, Matthew Welland, Eleanor Williams, Katarzyna Witkowska, and Suzanne Wood.

The members of the CORGI Consortium are Kai Ren Ong, Andrew Beggs, Alan Donaldson, Carole Brewer, Jayantha Arnold, Munaza Ahmed, Louise Izatt, Andrew Latchford, Dorothy Halliday, Peter Risby, Paul Brennan, Alison Kraus, Julian Barwell, Lynn Greenhalgh, D. Gareth Evans, Kate Green, Timothy Simmons, Rachel Harrison, Ragunath, Brian Davidson, Zoe Kemp, Helen Hanson, Katie Snape, Anneke Lucassen, Kevin J. Monahan, and Patrick Morrison.

The members of WGS500 Consortium are Peter Donnelly, John Bell, David Bentley, Gil McVean, Peter Ratcliffe, Jenny Taylor, Andrew Wilkie, John Broxholme, David Buck, Jean-Baptiste Cazier, Richard Cornall, Lorna Gregory, Julian Knight, Gerton Lunter, Ian Tomlinson, Andrew Wilkie, Christopher Allan, Moustafa Attar, Angie Green, Lorna Gregory, Sean Humphray, Zoya Kingsbury, Sarah Lamble, Lorne Lonie, Alistair Pagnamenta, Paolo Piazza, Amy Trebes, John Broxholme, Richard Copley, Simon Fiddy, Russell Grocock, Edouard Hatton, Chris Holmes, Linda Hughes, Peter Humburg, Alexander Kanapin, Stefano Lise, Hilary Martin, Lisa Murray, Davis McCarthy, Andy Rimmer, Natasha Sahgal, Ben Wright, and Chris Yau.

## Author contributions

Study supervision: C.P., J.R.S., I.P.M.T., I.J.M., and R.M.d.V. Analysis and drafting: C.P., E.C., J.E.G., S.G., C.F., H.W., J.R.S., I.P.M.T., I.J.M., and R.M.d.V. Data support: E.A.M.J., H.C., L.C., E.A.B., N.L., A.B., R.T., J.P., K.S., L.M., H.T., D.G., F.F., Y.G., D.J.A., S.A.M.v.d.B., M. Christie, M. Clendenning, L.E.T., C.D., J.A.D., D.D., J.L., K.M., R.S.v.d.P., M.S., J.W., J.C.T., C.T., L.V., T.v.W., C.W., C.A., G.C., W.C., D.C., A.F., A.G., B.K., B.N., D.C., T.G., R.H., N.L.B., A.S., D.W., M.A.J., R.P.K., A.W.R., J.P.C., M.J.L.L., N.H., V.H.K., A.D.R., I.M.W., C.R.P., D.D.D., D.G.P., and A.G. Critical revision: all authors. Shared last authors: J.R.S., I.P.M.T., I.J.M., and R.M.d.V.

## Data Availability

The WES data from WEHI-2 and D:II-1 datasets generated during this study are available at EGA (EGA: S00001004842 and EGA: S00001005063, respectively) after completion of a data transfer agreement. The WES data from CRDFF-292 supporting the current study have not been deposited in a public repository because of informed consent restrictions but are available from the corresponding author on request. WES/WGS data for ACCFR in the paper is available from the Colon Cancer Family Registry (https://www.coloncfr.org). Somatic variants in selected driver genes are available in Table S4. Somatic variant calls are available from the corresponding author on request.

## Web resources

Online Mendelian Inheritance in Man, http://www.omim.org

## Supplemental information

Data S1. Tables S2, S4, and S5

Document S2. Article plus supplemental information
