# Supplementary material for: Germline MBD4 deficiency causes a multi-tumor predisposition syndrome
Source: Am J Hum Genet. 2022 Apr 22;109(5):953–60. doi: 10.1016/j.ajhg.2022.03.018 (PMC9118112; doi:10.1016/j.ajhg.2022.03.018)
Supplement: Document S1. Supplemental methods, Figures S1–S3, and Tables S1 and S3 [file mmc1.pdf]

The American Journal of Human Genetics, Volume *109*

Supplemental information

Germline MBD4 deficiency causes a multi-tumor predisposition syndrome

Claire Palles, Hannah D. West, Edward Chew, Sara Galavotti, Christoffer Flensburg, Judith E. Grolleman, Erik A.M. Jansen, Helen Curley, Laura Chegwidden, Edward H. Arbe-Barnes, Nicola Lander, Rebekah Truscott, Judith Pagan, Ashish Bajel, Kitty Sherwood, Lynn Martin, Huw Thomas, Demetra Georgiou, Florentia Fostira, Yael Goldberg, David J. Adams, Simone A.M. van der Biezen, Michael Christie, Mark Clendenning, Laura E. Thomas, Constantinos Deltas, Aleksandar J. Dimovski, Dagmara Dymerska, Jan Lubinski, Khalid Mahmood, Rachel S. van der Post, Mathijs Sanders, Jürgen Weitz, Jenny C. Taylor, Clare Turnbull, Lilian Vreede, Tom van Wezel, Celina Whalley, Claudia Arnedo-Pac, Giulio Caravagna, William Cross, Daniel Chubb, Anna Frangou, Andreas J. Gruber, Ben Kinnersley, Boris Noyvert, David Church, Trevor Graham, Richard Houlston, Nuria Lopez-Bigas, Andrea Sottoriva, David Wedge, Genomics England Research Consortium, The CORGI Consortium, WGS500 Consortium, Mark A. Jenkins, Roland P. Kuiper, Andrew W. Roberts, Jeremy P. Cheadle, Marjolijn J.L. Ligtenberg, Nicoline Hoogerbrugge, Viktor H. Koelzer, Andres Dacal Rivas, Ingrid M. Winship, Clara Ruiz Ponte, Daniel D. Buchanan, Derek G. Power, Andrew Green, Ian P.M. Tomlinson, Julian R. Sampson, Ian J. Majewski, and Richarda M. de Voer

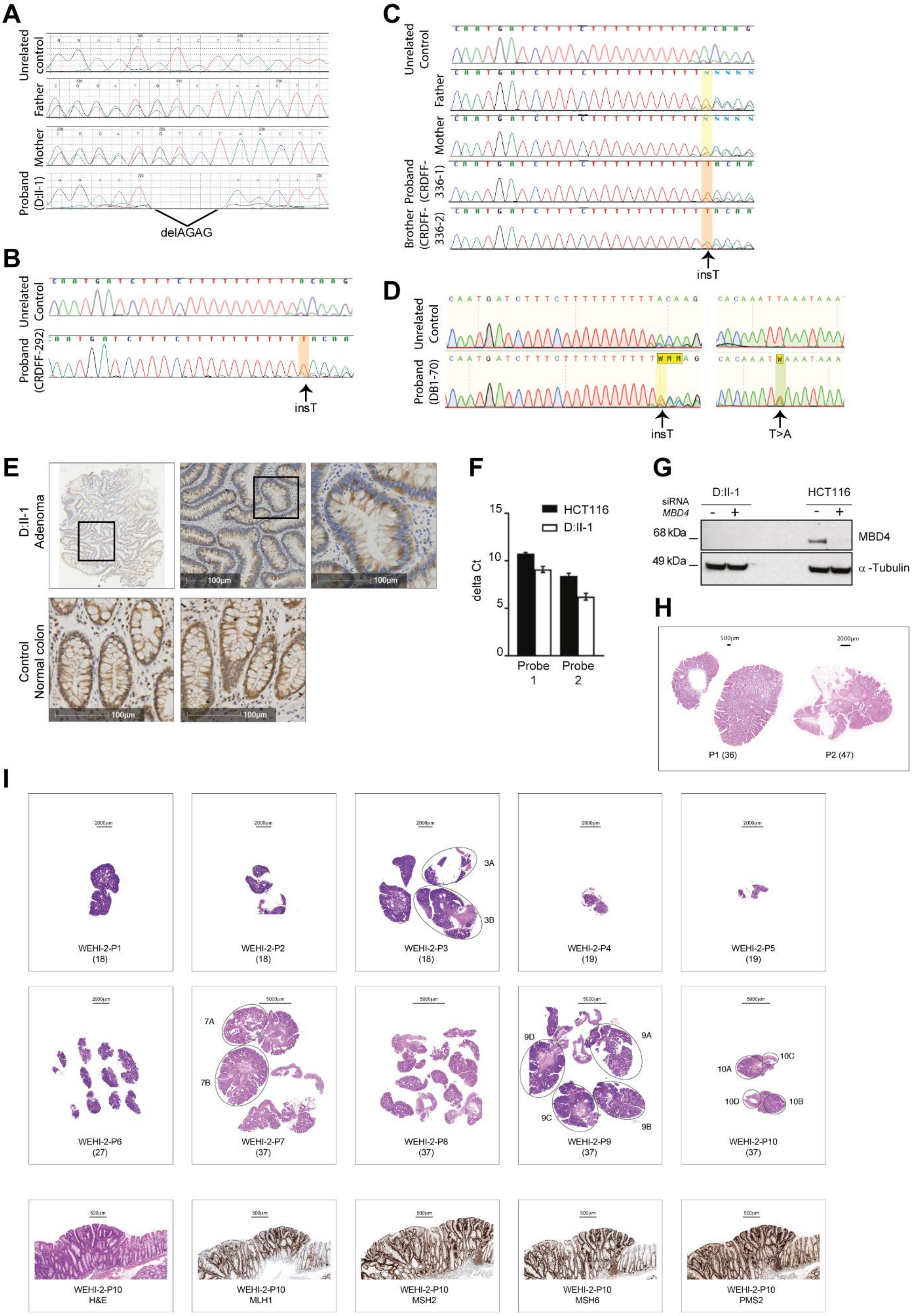

Figure S1: Sanger validations and *MBD4* expression in lymphoblastoid cells and histology of polyps from MBD4-deficient individuals. A) Sanger validations on genomic

DNA of the homozygous MBD4 loss-of-function (c.612_615del) variant in D:II-1 and parents. **B)** Sanger validations on genomic DNA (antisense strand) of the homozygous *MBD4* loss-of-function (c.939dup) variant in CRDFF-292 and unrelated control. Highlighted in orange the homozygous thymine insertion in CRDFF-292. **C)** Sanger validations on genomic DNA (antisense strand) of the homozygous *MBD4* loss-of-function (c.939dup) variant in CRDFF-336-1, parents, sibling and unrelated control. Highlighted in yellow the heterozygous thymine insertion in the parents of CRDFF-336-1. Highlighted in orange the homozygous thymine insertion in CRDFF-336-1 and her sibling CRDFF-336-2. **D)** Sanger validations on genomic DNA (antisense strand) of the heterozygous *MBD4* loss-of-function variants c.939dup (left) and c.1688T>A (right) variant in DB1-70 and unrelated control. Highlighted in yellow and green are the heterozygous thymine insertion and the heterozygous T>A change in DB1-70, respectively. **E)** Representative MBD4 IHC of an adenoma from simplex case D:II-1 (upper panels) and of a normal colon with wild type MBD4 (lower panels) stained with anti-MBD4 antibody. The D:II-1 (upper panels) show a tubular adenoma with low grade dysplasia showing typical nuclear changes (pencil shaped nuclei, crowding and pseudostrastification). **F)** RNA expression analysis showed stable expression of MBD4 as determined using two Taqman probes targeting MBD4 RNA (probe1=hS01023548; probe 2=HS00187498). Ct averages were 29.1 and 29.3 for HCT116 and D:II-1 respectively, using probe_1 and 26.7 and 26.4 for HCT116 and D:II-1 respectively, using probe_2. Bars are plotted as average of triplicate experiments with standard deviation. **G)** Western blot analysis of MBD4 expression in lymphoblastoid cells from simplex case D:Il-1 (-) and treated with an siRNA targeting MBD4 (+). The colorectal cancer cell line HCT116 was used a positive control. Two central lanes were left empty. Anti-alpha-Tubulin was used as loading control. **H)** Haematoxylin and eosin staining of polyps from D:II-1 with age in brackets. **I)** Haematoxylin and eosin staining and immunohistochemistry in polyps from WEHI-2. Rows 1 & 2: Haematoxylin and eosin (H&E) staining of sections from 10 polyps excised from WEHI-2, with age in years in brackets. Polyps P3, P7, P9 and P10 had multi-regions independently sequenced these regions are indicated by circles. Size bars represent 2000uM (P1 to P6) or 5000um (P7 to P10). Row 3: Immunohistochemistry staining of WEHI-2 P10 showing proficiency in mismatch repair proteins (MLH1, MSH2, MSH6 and PMS2). An H&E slide is shown for comparison. Size bars represent 500uM.

A

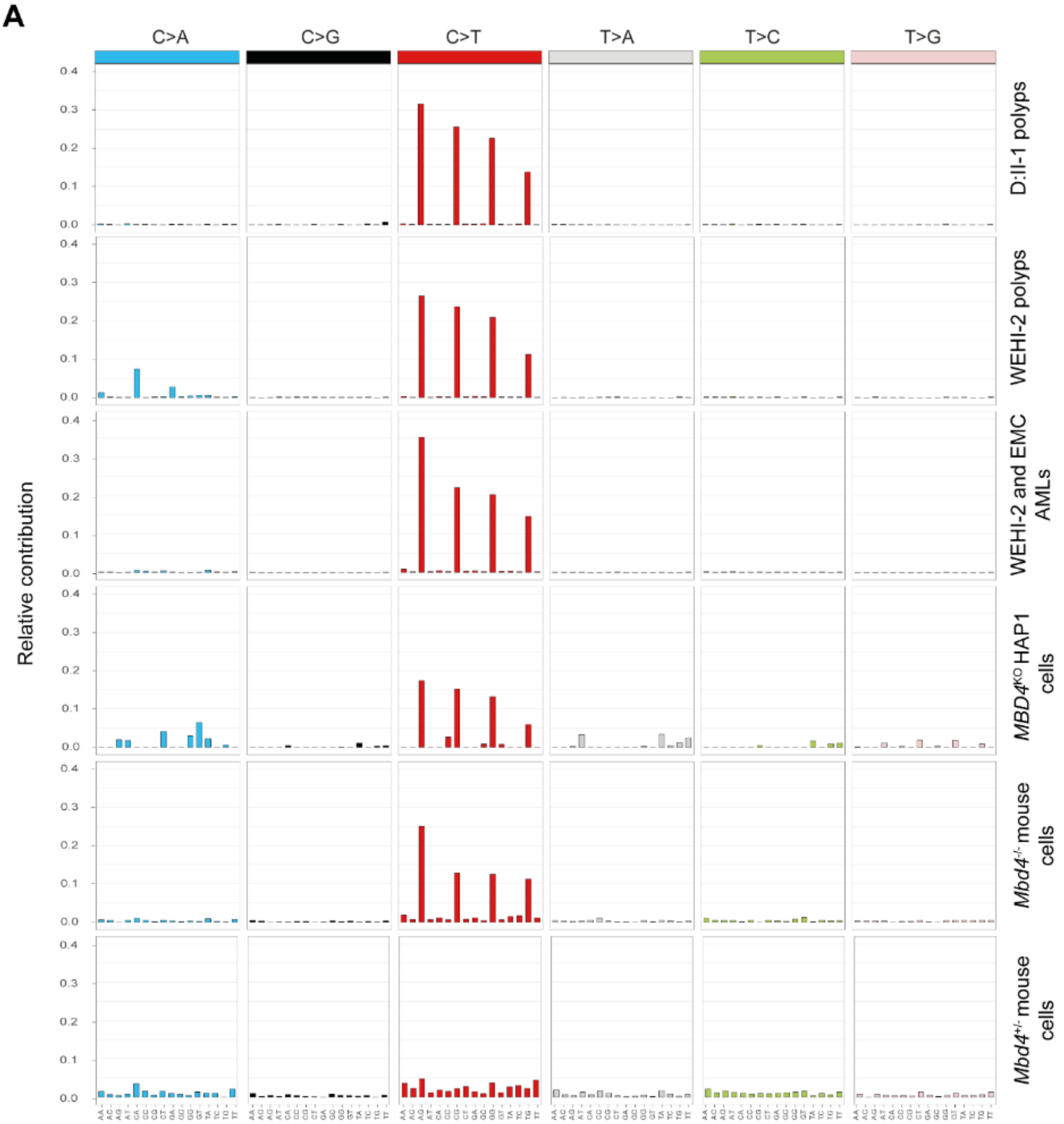

B

Mutational profiles	D:II-1	WEHI-2
SBS1-v3 (COSMIC)	0.9836	0.9602
WEHI and EMC AMLs	0.9928	0.9708
MBD4KO HAP1 cells	0.9683	0.9466
MBD4 -/- mouse cells	0.9193	0.9094
MBD4 +/- mouse cells	0.4867	0.5262

Figure S2: Mutation profiles and cosine similarity of observed mutations in various samples. A) Combined mutation profiles in the 96-mutation spectrum plot for each of the samples indicated. AML data originates from Sanders et al.[1] **B)** Cosine similarity scores indicate the closeness of the mutation profile of D:II-1 and WEHI-2 with the various mutations profiles observed in the other sequenced samples.

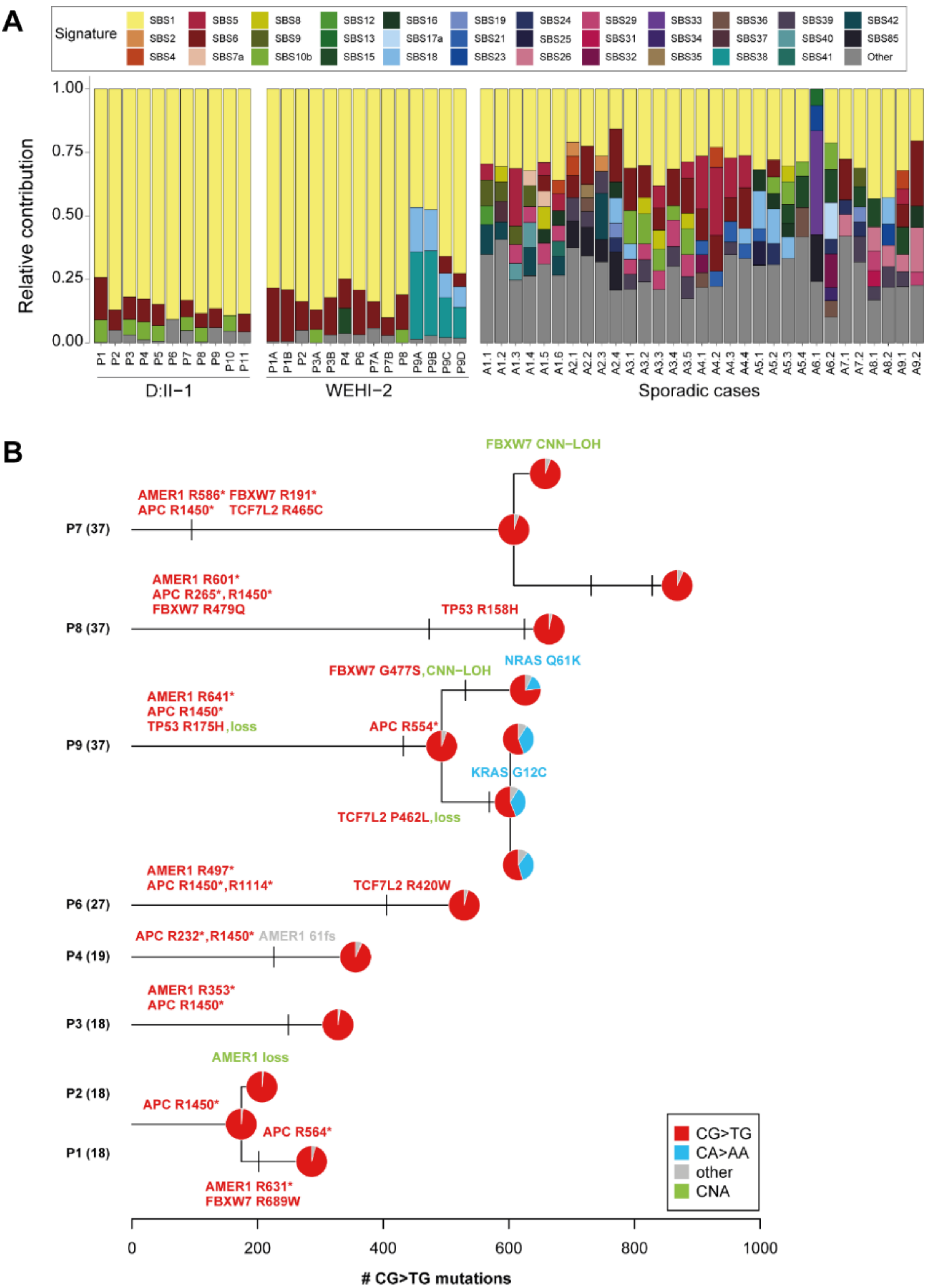

Figure S3: Mutational signature refitting of polyps of MBD4 deficient individuals and clonal evolution of polyps in WEHI-2. A) The estimated relative contribution of all known COSMIC-v3 mutational signatures to mutations in whole-exome sequenced adenomas from two MBD4-deficient individuals and nine adenomas from sporadic origin. For WEHI-2 and the sporadic polyps multi-regions were sequenced. For WEHI-2 multi-region sequenced polyps

are indicated by P1A or P1B, ect., and for the sporadic polyps this is indicated by by A1.1 and A1.2, ect. (see also Supplemental Table 2). Signatures with a contribution of less than 5% were merged into “other”. **B)** Clonal evolution of polyps in WEHI-2. These trees represents the development of clones within each polyp, with vertical bars or branches representing subclones. Note that P1 and P2 share a common precursor; labels are placed adjacent to the dominant clone in each polyp. The x-axis shows the number of somatic CG>TG mutations in each clone. Timing for key driver mutations is shown with earlier mutations on the left and the colour reflecting the type of mutation, either CG>TG (red), CA>AA (blue), copy number (green) or other (grey). All four physically distinct regions of WEHI-2 had a lower percentage of CpG>TpG transitions (55-77%) than the other adenomas (see also panel A) and a substantial proportion of mutations were CA>AA transversions (Figure 2C). The different regions were clonally related and, using CpG>TpG transitions as a molecular clock, we found that this shift in mutational profile likely coincided with treatment for AML.

Table S1: Patient cohort inclusion and results of the targeted *MBD4* screening

**Cohort**	**# samples**	**Selection Criteria**	**Genes tested negative**	**Loss of function germline variants in *MBD4***
Skopje, Macedonia	12	Polyposis, recessive inheritance	MMR genes, *APC*, *TP53*, *MUTYH*, *POLE*, *POLD1*	none
Nijmegen, the Netherlands	147	Polyposis or familial CRC	*APC*, *MUTYH*	none
Leiden, the Netherlands	149	Polyposis or familial CRC	*APC*, *MUTYH*	none
Dresden, Germany	100	Polyposis or familial CRC	*APC*, *MUTYH*	none
Oxford, United Kingdom	275	Polyposis	*APC*, *MUTYH*	c.1437G>A; heterozygous (n = 1)<br>c.1636C>T; heterozygous (*n* = 1)
Szczecin, Poland	144	Familial CRC	*POLE*, *POLD1*, MMR genes*	none
Santiago de Compostela, Spain	35	Polyposis or familial CRC	*APC*, *MUTYH* (partly), *POLE*, *POLD1, BMPR1A, SMAD4, PTEN*	c.1562-1G>T; heterozygous (*n* = 1)
ParelBED, the Netherlands	600	Polyposis with or without CRC, and/or CRC in combination with (a) different tumour(s)	No disease causing mutation found (tested negative or not tested)	c.1410A>C; heterozygous (*n* = 1)
CORGI collaborator, Greece	28	polyposis (10-100 polyps) with or without CRC	*APC, MUTYH, NTHL1 (NTHL1 tested in most samples and negative in all tested)*	none
CORGI collaborator, Cyprus	2	polyposis	unknown	none
CORGI collaborator, Australia	1	polyposis	unknown	none
Cardiff, England	118	polyposis	*APC, MUTYH*	c.939dup; homozygous (*n* = 1)
**Screening Total**	1,611			

MMR genes: MLH1, MSH2, MSH6, and PMS2. *Most patients were tested for these genes.

Table S3: Enrichment analysis of *MBD4* loss of protein function variants in individuals with polyposis and/or CRC and/or AML/MDS

Cohort (*n*)	Cases (*n*)	Controls (*n*)	OR	95% CI	*P*-value
**Replication study**					
polyposis and/or CRC (1,611)	4	na	0.3	0.11-1.14	0.038
gnomAD (64,600)	na	48	-	-	-
**UK Biobank**					
CRC (2,357)*	2	na	1.02	0.28-8.48	1
polyposis (5,116)*	1	na	4.42	0.78-175.70	0.138
AML (221)*	1	na	0.19	0.03-7.59	0.175
MDS (104)*	1	na	0.09	0.02-3.57	0.086
others (193,255)	na	167	-	-	-
**100KGP**					
CRC (2,438)#	4	na	0.74	0.25-2.98	0.542
others (17,243)	na	21	-	-	-

* Fisher's exact test compared with others in UK Biobank
# Fisher's exact test compared with others in 100KGP
Abbreviations: 100KGP: 100,000 genomes project; OR: odds ratio; CI: confidence interval; AML: acute myeloid leukaemia; MDS: myelodysplastic syndrome

Supplementary Methods:

Study cohorts and whole-genome and -exome sequencing for germline variants

i) Individuals with multiple colorectal adenomas were recruited via the "Identification and characterization of Inherited Predispositions to Colorectal Tumors" (UK CORGI) study. As part of the Oxford-Illumina WGS500 project, we performed whole-genome sequencing of constitutional DNA extracted from peripheral blood lymphocytes from 35 individuals with at least 10 colorectal adenomas before age 60 who tested negative for polyposis- and colorectal cancer predisposing genes in routine diagnostics.[2] The Illumina HiSeq platform was used and a median of ~40X coverage achieved. Read alignment and variant calling were performed using BWA, Stampy and Platypus as described.[2] Samples were additionally joint called using GATKv3 and annotated using ANNOVAR.[2] We extracted all variants predicted to result in protein truncation (nonsense, frameshift, or splice site variants) and prioritized for homozygous and compound heterozygous variants with a low frequency (MAF < 0.01) in the general population.

ii) Individuals (n = 74) with 10 or more colorectal adenomas with or without colorectal cancer who had been referred to specialist clinical genetics services for investigation of a possible inherited polyposis syndrome, but in whom genetic testing including *APC* and *MUTYH* had not revealed a genetic cause were recruited after giving informed consent to participate in the 'Genetic Mechanism in Polyposis of the Bowel' study

(REC 12/WA/0071). Constitutional DNA was extracted from peripheral blood lymphocytes. Whole-exome libraries were prepared using the TruSeq DNA Exome kit (Illumina) according to the manufactures instructions. Libraries were normalized, pooled and sequenced using a 75-base paired-end dual index read format on the Illumina HiSeq4000 according to the manufacturer's instructions. Read alignment and variant calling were performed using BWA against GRCh37h (1000 genomes version human_g1k_v37.fasta). BAM files were subjected to post-processing using samtools (sorted, fixmate, rmdup) to fix mate pairs and remove duplicates. Variants were called using GATK HaplotypeCaller on the recalibrated bam files. Variants were annotated using VEP, outputting all available annotation data. We extracted variants predicted to result in protein truncation (nonsense and frameshifting) and prioritized for homozygous and compound heterozygous variants with a low frequency (MAF <1%) in the general population.

iii) Colorectal cancer affected families with blood and tumor specimens available were selected for WGS and WES from the Australasian Colorectal Cancer Family Registry (ACCFR). Families with a pathogenic variant within a known hereditary CRC and polyposis susceptibility gene were excluded. Eighty-seven families comprised of 198 CRC- and early-onset polyp affected people were selected for sequencing based on having a family history of CRC that met one of the following criteria: 1) met the definition of Familial Colorectal Cancer Type X (FCCTX; *n* = 55), 2) Amsterdam II clinical criteria (AMII) with 2 of the defining triad being CRC-affected (n = 4), 3) >2 CRC-affected family members within the same blood line but not meeting FCCTX or AMII criteria (MCF; *n* = 28). Written informed consent was obtained from all study participants and the study protocol approved by Human Research Ethics Committees at the University of Melbourne (HREC#1954921). Germline whole exome sequencing (WES) was performed using SureSelect Human All Exon V4 (51Mb) kit (Agilent, Santa Clara, CA, USA) and 100bp paired-end sequencing on the Illumina HiSeq2000 to a mean coverage of 100x (Macrogen Inc., South Korea). Whole genome sequencing (WGS), using Illumina TruSeq DNA sample preparation and 100bp paired-end sequencing on the Illumina HiSeq2000 to a mean coverage of 30x (Macrogen Inc., South Korea). Sequence reads were mapped to the Human Reference Genome GRCh37 using BWA (v 0.7.12). Germline single nucleotide variants (SNVs) and short insertions and deletions (INDELs) were calculated using the GATK best practices pipeline (v 4.0.0) and we extracted variants predicted to result in protein truncation (nonsense and frameshifting) and prioritized for homozygous and compound heterozygous variants with a low frequency (MAF <1%) in the general population.

UK 100,000 genomes samples (100KGP) - We searched the genome sequencing data available (ISAAC pipeline) for 17,243 Caucasian participants of the rare diseases programme (v6 release, participants selected for phenotypes with no increased risk of cancer), 2,438 Caucasian CRC individuals included in the cancer programme (v8 release) and 283 Caucasian individuals with multiple bowel polyps (143 v6 rare diseases, 140 pilot project) for germline coding or splicing variants in *MBD4*. Variants were prioritized as described below.

UKBiobank samples - We searched the exome and genome sequencing data available for 200,000 participants in UKBiobank (exome sequencing; October 26th 2020 release) for germline coding or splicing variants in MBD4. This research was conducted under UK Biobank application number 8508. Variants were prioritized as described below.

GnomAD - We searched the full and non-Finnish European populations of the gnomAD database (v2.1 dataset) for germline coding or splice site variants in *MBD4*. Variants were prioritized as described below.

Molecular inversion probe sequencing of *MBD4* - All participants in the MIP screen provided written informed consent. Details of the inclusion criteria are described in **Table S1**. This study was approved by the local medical ethics committee (CMO light; study number 2015/2172 of the Radboudumc Nijmegen). Leukocyte-derived DNA was used for targeted resequencing of *MBD4* (NM_003925.2) using 32 Molecular Inversion Probes (MIPs), covering all coding regions and intron-exon boundaries, were designed according to the previously published methodology.[3,4] After targeted capture, samples were sequenced on a NextSeq500 (Illumina) system. Reads were mapped using BWA and variants called using GATK's UnifiedGenotyper. After variant calling, all variants with an at least 40-fold absolute coverage, ≥20 variant reads, ≥25% variant reads and ≥8.000 quality by depth scores were selected for further analyses. Loss of function (LOF) variants in *MBD4* (see below) identified using MIP-sequencing with a quality by depth score of 8.000-11.000 were validated using Sanger sequencing.

Sanger sequencing of *MBD4* - Bidirectional Sanger sequencing of the *MBD4* open reading frame and flanking intronic regions was undertaken in blood DNA samples from 118 individuals from the UK 'Genetic Mechanisms in Polyposis of the Bowel' study who were not included in the WES screening for germline pathogenic variant. All had at least 10 colorectal adenomas and had tested negative for pathogenic variants in *APC* and *MUTYH* in an NHS diagnostic setting. Primer design (available upon request) was carried out using Primer3 and primers checked for template (NG_033106.1) specificity using Primer BLAST. PCR was carried out using MegaMix Gold (Microzone). PCR products were clean-upup using exonuclease (New

England Biolab) and shrimp alkaline phosphatase (ThermoFisher) and subjected to Sanger sequencing on an ABI 3730 analyser (Applied Biosystems). Sanger chromatograms were visualised with Sequencher (Gene Codes, USA).

MBD4 variant interpretation - All *MBD4* variants located in an exon region, canonical splice site (positions +1, +2 and −2, -1), and coding or noncoding splice site region (3' splice site -12 till +2 and 5' splice site -3 till +6) were included for further analyses. Furthermore, only variants with an allele frequency <1% in an in-house database of 12,244 germline exomes that have been sequenced at Genome Diagnostics Nijmegen (https://order.radboudumc.nl/en/genetics) and <2% in ExAC and gnomAD were included. To select variants of pathogenic potential, we selected all frameshift and nonsense variants, and missense variants with a PhyloP score ≥3 and a CADD_PHRED score ≥15. For variants with a predicted splicing effect of more than 20% by SpliceAI additional in silico splice site predictions were obtained using MaxEntScan, NNSPLICE, and Human Splicing Finder (Alamut Visual 2.13). Splice site losses were included when 1) the variant splice score was less than 50% of the scoring range for at least two algorithms and 2) the difference between wildtype and variant splice score was more than 20% of the scoring range in at least three algorithms. Splice site gains were included when 1) the variant splice score was above 75% of the scoring range for at least two algorithms and 2) the difference between the gained splice site and the nearest splice site was more than 2% of the scoring range in at least two algorithms.

Whole-exome sequencing of adenomas - WEHI-2 (previously reported as WEHI-AML-2) consented to the use of their clinical information and tissues for research in accordance with the Declaration of Helsinki. The project was approved by human research ethics committees from the Walter & Eliza Hall Institute of Medical Research (WEHI) and Melbourne Health (MH) (WEHI HREC project 13/01, MH HREC project 2012.274). DNA was extracted from nine fresh frozen adenomas, two formalin fixed paraffin embedded (FFPE) adenomas and three fresh frozen macroscopically normal bowel tissues from D:II-1, nine independent FFPE adenomas from WEHI-2 **(Figure S1H-I; Table S2)**. Exome library preparations were performed according to the manufacturer using either the i) Agilent SureSelectXT Human All Exon V6 (Agilent Technologies), ii) Agilent SureSelect XT Low Input Human Whole Exome V6 (Agilent Technologies) or iii) Illumina TruSeq exome (Illumina) enrichment kit in combination with sequencing on a NextSeq500 (Illumina) or NovaSeq 6000 (Illumina) **(Table S2)**.

WES sequencing reads from D:II-1 were aligned with BWA and stampy v1.0.28. Duplicates were marked using picard 1.9.2. Clonal tracking was performed with default superFreq (v1.3.2)[5] using preliminary variants from VarScan (v2.3)[6] with the options --strand-filter 0, --p-value 0.05 and --min-var-freq 0.02. For the FFPE samples >700,000 variants were

called per sample using these settings compared to ~100,000 in the fresh frozen samples. Varscan VCFs from FFPE samples were additionally filtered to remove variants with <10% VAF and <4 reads supporting the variant allele. Somatic variants were called using Mutect2 (GATK version 4.1.0.0). Normal bowel samples from the caecum and transverse colon of simplex case D:II-1 were used as matched normal, a panel of 6 normal colon samples from other persons without CRC or polyps were used as a panel of normals reference to assist with filtering platform artefacts. “af-only-gnomad.raw.sites.b37.vcf” was provided as an additional germline reference.

WES sequencing reads from WEHI-2 were aligned to hg19 with BWA[7] Variant calling, CNA calling and clonal tracking was done with default superFreq (v1.3.2)[5] using preliminary variants from VarScan (v2.3)[6] with the options --strand-filter 0, --p-value 0.01 and --min-var-freq 0.05. To limit artefacts, the variants were further filtered based on clonal assignment.

For each adenoma, high confidence somatic mutations were identified as described previously with minor modifications.[8] In brief, somatic variants covered by ≥15x sequencing reads, ≥10% variant allele frequency, and ≥6 variant reads, and (for variants called by Mutect2) with ≥2 variant reads per read pair (to exclude FFPE artefacts) were included. WEHI-2 received an allogeneic bone marrow transplant and variants contributed by the haematopoietic stem cell donor were removed based on clonal tracking in SuperFreq.[5] For somatic variants identified by SuperFreq, a mean read depth >30 across all samples was required and excluded variants if they were detected above 3% VAF (supported by at least 2 reads) in a sample where the clone was deemed absent (clonality <1%). A representative set of somatic mutations were validated by Sanger sequencing or by processing micro-dissected material from the adenomas with the TruSight Tumor 26 Kit (Illumina), including one additional adenoma from WEH-2 (see also **Table S2**).

WES methods for the sporadic adenoma samples was previously described.[9] Somatic variants were called from the BAMs for each region, which were previously aligned to hg19, using Mutect2, following the same strategy as described for D:II-1. Each adenoma sample had a matched normal. In all analyses the union of mutations called in any of the regions from the same adenoma were combined.

Somatic mutation spectrum and driver gene analyses - The number of somatic mutations per megabase (Mb), mutation spectrum and the number of CpG>TpG transitions was determined. A linear model describing the number of CpG>TpG transitions as a function of age was analyzed in base R. Methylation status of the sites with somatic mutation was assessed in public whole genome bisulfite sequencing (WGBS) data from normal sigmoid colon from the Roadmap Epigenomics Consortium.[10] The contribution of mutational signatures to the somatic

mutation spectrum was inferred using the R package MutationalPatterns[11] in combination with COSMIC-v3 mutational signatures.

To compare to the The Cancer Genome Atlas (TCGA) CRC data[12], we downloaded variant calls from SomaticSniper, VarScan2, MuTect2, and MuSE through the National Cancer Institute Genomic Data Commons. Variants were retained if the variant allele frequency was greater than 20% with at least 20 read depth, and if it was identified by at least 3 of the 4 callers. Mismatch repair status was available for a subset of samples, which we used to classify tumors as microsatellite stable (MSS) or unstable (MSI). All non-synonymous somatic variants in cancer driver genes reported by The Cancer Gene Census (CGC v92) in COSMIC were extracted for each of the sequenced adenomas. Next, driver genes were prioritised with their previous associations as a colorectal cancer driver by TCGA and Dietlein et al.[12,13] To compare driver genes and mutation types, genes that were mutated significantly different in the MBD4-deficient individuals or sporadic adenomas were plotted in an oncoprint.

CRISPR/Cas9 generated *MBD4* knockout cells - HAP1 cells were maintained in Iscove's Modified Dulbecco's Medium (IMDM; GIBCO), containing 10% fetal calf serum (FCS), 1% glutamine, and 1% penicillin/streptomycin. Single guide RNAs (sgRNA) targeting the glycosylase domain of *MBD4* were designed using CHOPCHOP, cloned into the Cas9 expression vector PX459 v2.0 (Addgene plasmid #62988) and HAP1 cells were transfected according to the method described by Ran *et al.*[14] with minor modifications. After puromycin selection single cell clones were derived using a FACS sorting. Effective knockout of *MBD4* was determined based on Sanger sequencing of the target regions, mRNA expression and by Western blot. Full *MBD4* knockout (*MBD4*^{KO}) HAP1 single cell clones were cultured for 142 days, followed by another single cell sort. Subclones were expanded for 14 days and gDNA was isolated from the parental HAP1 clone and *MBD4*^{KO} subclones. Two WT and *MBD4*^{KO} clones were whole-genome sequenced using the TruSeq DNA PCR-Free library kit (Illumina) and sequenced on a NovaSeq 6000 System (Illumina). Reads were mapped using BWA and for all samples at least 90% of the genome was covered at 20X. Variant calling and mutational signature analysis was performed as described above. As modification to the analysis the average 96-profile of the HAP1 WT clones was extracted from the *MBD4*^{KO} clone to be left with the 96-profile specific to the *MBD4* knockout.

Assessment of mutation rate in a mouse model of MBD4 deficiency - Whole genome sequencing was performed on individual mouse haematopoietic progenitor colonies as previously described.[1] In brief, mouse bone marrow cells were cultured in semi solid agar. Each culture contained 10,000 bone marrow cells, suspended in Dulbecco modified Eagle medium with 20% bovine calf serum and 0.3% agar, with 100ng murine stem cell factor, 10ng

murine IL-3 and 2 IU erythropoietin. Cells were incubated for 11 days at 37°C in a humidified atmosphere with 10% CO_2. DNA was extracted from individual colonies using QIAamp DNA Micro Kit (Qiagen), amplified using TruePrime WGA Kit (SYGNIS) and purified using QIAamp DNA Mini Kit (Qiagen). Whole genome sequencing was performed on the NovaSeq 6000 (150bp paired end reads, Illumina). The mouse sequencing data was aligned to the mouse genome (mm10) using bwa-mem. WGS was also performed on the original bone marrow DNA and used to identify variants unique to the individual colony. Results from the wildtype and knockout colonies were reported previously and were deposited at SRA (Accession: PRJNA419992).[1]

Generation of a lymphoblastoid cell line from simplex case D:II-1 - Peripheral blood lymphocytes (PBLs) were isolated using Ficoll-Paque PLUS (eppendorf) following manufacturers instructions from a fresh blood sample, collected in sodium heparin tubes from simplex case D:II-1. A lymphoblastoid line was generated by Epstein Barr virus transformation by the Culture collections team, Public Health England.

RNA and protein analysis - Taqman expression probes HS01023548 and HS00187498 were used to quantify *MBD4 m*RNA extracted from a lymphoblastoid cell line from simplex case D:II-1 (further details available upon request). Protein lysates from cells were analysed by western blotting using anti-MBD4 antibody ab224809 (Abcam). HCT116, HAP1 and D:II-1 lymphoblastoid cells were resuspended in RIPA buffer (Thermo Fisher Scientific). Total lysates were quantified with Pierce BCA Protein Assay kit (Thermo Fisher Scientific) according to manufacturer’s instructions. 20 µg of protein lysate were loaded on a 4-20% gradient gel (Thermo Fisher Scientific) or NuPage 4-12% Bis-Tris Gels (Invitrogen). After transfer with iBlot2 dry Blotting System (Thermo Fisher Scientific) and blocking membranes were blotted for anti-MBD4 (abcam, diluted 1:1000) and anti-α-tubulin (Sigma, 1 diluted 1:5000 or Abcam, diluted 1:500) as a loading control. Membranes were exposed to hyperfilm ECL (GE Healthcare) and developed using a X-Ray Film Processor (Konica) or scanned on the Odyssey Infrared Imaging System (Li-COR).

MBD4 immunohistochemistry - Formalin-fixed paraffin-embedded (FFPE) specimens of colorectal adenomas were analysed by immunohistochemistry using the same anti-MBD4 antibody. Samples sections (5 µm) were deparaffinized with xylene and rehydrated. Sections were incubated with 6% H_2O_2 for 20 min at room temperature to block endogenous peroxidase activity. Antigen retrieval was carried out by incubating the slides in citrate buffer (pH 6.0) at 95°C for 10 min. Sections were blocked with goat serum for 30 min at room temperature. Primary antibodies were diluted in 1% goat serum/0.1% BSA/PBS.

Sections were incubated with MBD4 primary antibody (ab224809, 1:50) overnight at 4°C. Sections were washed with Tris-buffered saline with 0.1% Tween 20 (TBST) and incubated with secondary antibody anti rabbit (PK-6100) for 30 min. Tertiary (ABC Biotinylated) was kept for 30 min. Staining was visualized using a HRP/DAB detection system Dako. Control IHC experiments (data not shown) were performed without primary antibody. All sections were counterstained with Gill's haematoxylin and mounted for digital slide scanning using a Zeiss ActioScan Z1.

Acknowledgements: We thank all families for their collaboration. We thank Dr. Robbert D.A. Weren, Dr. Janet Vos and Eveline Kamping for technical assistance, the Genome Technology Platform for MIP sequencing support, Dr. Christian Gillissen for the use of the annotation pipeline in the Radboudumc, Nijmegen, and Dr. Peggy Manders for the use of samples from the Dutch Parelsnoer Institute Biobank Hereditary Colorectal Cancer. We thank expert colonoscopists in Cardiff, Drs Gareth Thomas and Sunil Dolwani. We acknowledge expert technical assistance from the Austin Molecular Laboratory and the Australian Genome Research Facility for providing expert technical assistance with genomic analysis of cancer samples. We thank the Wales Gene Park for expert help with WES and processing of data, Loys Richards and Laura Butlin for their expert assistance with research governance and Karen Bailey for help with study participant recruitment. We thank members of the Colorectal Oncogenomics Group and the participants and staff from the Colon-CFR in particular, Maggie Angelakos, Samantha Fox and Allyson Templeton for their support of this study. Computation and bioinformatics were also provided by Melbourne Bioinformatics on its Peak Computing Facility. Furthermore, we thank the Birmingham Genomics Service at The University of Birmingham, for the generation of the sequencing and methylation array data. Some of the computations described in this paper were performed using the University of Birmingham's BlueBEAR HPC service, which provides a High Performance Computing service to the University's research community. We thank all participants of the CORGI study for their collaboration. This research was made possible through access to the data and findings generated by the 100,000 Genomes Project. The 100,000 Genomes Project is managed by Genomics England Limited (a wholly owned company of the Department of Health and Social Care). The 100,000 Genomes Project is funded by the National Institute for Health Research and NHS England. The Wellcome Trust, Cancer Research UK and the Medical Research Council have also funded research infrastructure. The 100,000 Genomes Project uses data provided by study participants and collected by the National Health Service as part of their care and support.

REFERENCES

1. Sanders MA, Chew E, Flensburg C, et al. MBD4 guards against methylation damage and germ line deficiency predisposes to clonal hematopoiesis and early-onset AML. Blood 2018;132:1526-1534.
2. Palles C, Cazier JB, Howarth KM, et al. Germline mutations affecting the proofreading domains of POLE and POLD1 predispose to colorectal adenomas and carcinomas. Nat Genet 2013;45:136-44.
3. O'Roak BJ, Vives L, Fu W, et al. Multiplex targeted sequencing identifies recurrently mutated genes in autism spectrum disorders. Science 2012;338:1619-22.
4. Boyle EA, O'Roak BJ, Martin BK, et al. MIPgen: optimized modeling and design of molecular inversion probes for targeted resequencing. Bioinformatics 2014;30:2670-2.
5. Flensburg C, Sargeant T, Oshlack A, et al. SuperFreq: Integrated mutation detection and clonal tracking in cancer. PLoS Comput Biol 2020;16:e1007603.
6. Li H, Durbin R. Fast and accurate short read alignment with Burrows-Wheeler transform. Bioinformatics 2009;25:1754-60.
7. Koboldt DC, Zhang Q, Larson DE, et al. VarScan 2: somatic mutation and copy number alteration discovery in cancer by exome sequencing. Genome Res 2012;22:568-76.

8. Grolleman JE, de Voer RM, Elsayed FA, et al. Mutational Signature Analysis Reveals NTHL1 Deficiency to Cause a Multi-tumor Phenotype. Cancer Cell 2019;35:256-266
9. Cross W, Kovac M, Mustonen V, et al. The evolutionary landscape of colorectal tumorigenesis. Nature ecology & evolution 2018;2:1661-1672.
10. Roadmap Epigenomics Consortium; Kundaje A, Meuleman W, Ernst J, et al. Integrative analysis of 111 reference human epigenomes. Nature 2015;518:317-330.
11. Blokzijl F, Janssen R, van Boxtel R, et al. MutationalPatterns: comprehensive genome-wide analysis of mutational processes. Genome Med 2018;10:33.
12. Cancer Genome Atlas Network. Comprehensive molecular characterization of human colon and rectal cancer. Nature 2012;487:330-337.
13. Dietlein F, Weghorn D, Taylor-Weiner A, et al. Identification of cancer driver genes based on nucleotide context. Nat Genet 2020;52:208-218.
14. Ran FA, Hsu PD, Wright J, Agarwala V, Scott DA, Zhang F. Genome engineering using the CRISPR-Cas9 system. Nat Protoc. 2013;8(11):2281-2308.
