# Supplementary material for: Germline MBD4 deficiency causes a multi-tumor predisposition syndrome
Source: Am J Hum Genet. 2022 Apr 22;109(5):953–60. doi: 10.1016/j.ajhg.2022.03.018 (PMC9118112; doi:10.1016/j.ajhg.2022.03.018)
Supplement: Document S2. Article plus supplemental information [file mmc3.pdf]

Germline MBD4 deficiency causes a multi-tumor predisposition syndrome

Authors

Claire Palles, Hannah D. West, Edward Chew, ...,
Julian R. Sampson, Ian J. Majewski,
Richarda M. de Voer

Correspondence

ian.tomlinson@igmm.ed.ac.uk (I.P.M.T.),
sampson@cf.ac.uk (J.R.S.)

Palles et al., 2022, The American Journal of Human Genetics *109*, 953–960
May 5, 2022 © 2022 The Author(s).
https://doi.org/10.1016/j.ajhg.2022.03.018

CellPress

Germline MBD4 deficiency causes a multi-tumor predisposition syndrome

Claire Palles,[1,41] Hannah D. West,[2,41] Edward Chew,[3,41] Sara Galavotti,[1,41] Christoffer Flensburg,[3,41] Judith E. Grolleman,[4,41] Erik A.M. Jansen,[4] Helen Curley,[1] Laura Chegwidden,[1] Edward H. Arbe-Barnes,[5] Nicola Lander,[2] Rebekah Truscott,[2] Judith Pagan,[6] Ashish Bajel,[7] Kitty Sherwood,[8] Lynn Martin,[1] Huw Thomas,[9] Demetra Georgiou,[10] Florentia Fostira,[11] Yael Goldberg,[12,13]

(Author list continued on next page)

Summary

We report an autosomal recessive, multi-organ tumor predisposition syndrome, caused by bi-allelic loss-of-function germline variants in the base excision repair (BER) gene *MBD4*. We identified five individuals with bi-allelic *MBD4* variants within four families and these individuals had a personal and/or family history of adenomatous colorectal polyposis, acute myeloid leukemia, and uveal melanoma. *MBD4* encodes a glycosylase involved in repair of G:T mismatches resulting from deamination of 5′-methylcytosine. The colorectal adenomas from MBD4-deficient individuals showed a mutator phenotype attributable to mutational signature SBS1, consistent with the function of MBD4. MBD4-deficient polyps harbored somatic mutations in similar driver genes to sporadic colorectal tumors, although *AMER1* mutations were more common and *KRAS* mutations less frequent. Our findings expand the role of BER deficiencies in tumor predisposition. Inclusion of *MBD4* in genetic testing for polyposis and multi-tumor phenotypes is warranted to improve disease management.

Inherited defects in DNA repair are responsible for a group of genetic tumor risk syndromes that are characterized by adenomatous polyposis, colorectal cancer (CRC), and extracolonic neoplasms. These syndromes include dominantly inherited polymerase proofreading-associated polyposis (PPAP) caused by pathogenic variants in the polymerase proofreading domains of *POLE* (MIM: 615083) and *POLD1* (MIM: 612591)[1] and recessively inherited conditions caused by variants in genes involved in mismatch repair (*PMS2* [MIM: 614337], *MSH6* [MIM: 600678], *MSH2* [MIM: 120435], *MLH1* [MIM: 609310])[2,3] and base-excision repair (BER) (*MUTYH* [MIM: 608456] and *NTHL1* [MIM: 616415]).[4,5] Mechanistically, defective DNA repair appears to lead to an increase in the somatic mutation rate and accumulation of somatic mutations in cancer driver genes such as *APC* (MIM: 611731), *KRAS* (MIM: 190070), and *TP53* (MIM: 191170). Previous studies have linked the specific defects in DNA repair genes to mutational signatures.[6–9]

Genetic testing currently fails to identify a cause in a significant proportion of individuals who develop multiple colorectal adenomas. It is important to identify any

[1]Institute of Cancer and Genomic Sciences, College of Medical and Dental Science, University of Birmingham, Edgbaston, Birmingham B15 2TT, UK; [2]Institute of Medical Genetics, Division of Cancer and Genetics, Cardiff University, School of Medicine, Cardiff, UK; [3]Walter and Eliza Hall Institute of Medical Research, Parkville, VIC 3052, Australia; [4]Department of Human Genetics, Radboud Institute for Molecular Life Sciences, Radboud University Medical Center, 6525 Nijmegen, the Netherlands; [5]Wellcome Trust Centre for Human Genetics, University of Oxford, Oxford OX3 7BN, UK; [6]Molecular Genetics Laboratory, South East Scotland Genetic Service, Western General Hospital, Crewe Road, Edinburgh EH4 2XU, UK; [7]Peter MacCallum Cancer Center and Royal Melbourne Hospital, Victorian Comprehensive Cancer Centre, Parkville, VIC, Australia; [8]Edinburgh Cancer Research Centre, IGMM, University of Edinburgh, Crewe Road, Edinburgh EH4 2XR, UK; [9]St Mark's Hospital, Imperial College London, London, UK; [10]Genomic Medicine, Imperial College Healthcare Trust and North West Thames Regional Genetics Service, Northwick Park, Harrow, UK; [11]Molecular Diagnostics Laboratory, NCSR Demokritos, Athens, Greece; [12]Raphael Recanati Genetic Institute, Rabin Medical Center – Beilinson Hospital, Petach Tikva, Israel; [13]Sackler Faculty of Medicine, Tel Aviv University, Tel Aviv, Israel; [14]Wellcome Trust Sanger Institute, Wellcome Genome Campus, Hinxton, Cambridge CB10 1SA, UK; [15]Colorectal Oncogenomics Group, Department of Clinical Pathology, Melbourne Medical School, The University of Melbourne, Parkville, VIC, Australia; [16]University of Melbourne Centre for Cancer Research, Victorian Comprehensive Cancer Centre, Parkville, VIC, Australia; [17]Institute of Life Sciences, Swansea University, Swansea SA28PP, UK; [18]Center of Excellence in Biobanking and Biomedical Research and Molecular Medicine Research Center, University of Cyprus Medical School, Nicosia, Cyprus; [19]Center for Biomolecular Pharmaceutical Analyzes, UKIM Faculty of Pharmacy, 1000 Skopje, Republic of Macedonia; [20]Hereditary Cancer Center, Department of Genetics and Pathology, Pomeranian Medical University, 70-111 Szczecin, Poland; [21]Department of Pathology, Radboud Institute for Molecular Life Sciences, Radboud University Medical Center, 6525 Nijmegen, the Netherlands; [22]Department of Hematology, Erasmus University Medical Center, Rotterdam, the Netherlands; [23]Department of Surgical Research, Universitätsklinikum Carl Gustav Carus, Technische Universität Dresden, 01307 Dresden, Germany; [24]Oxford NIHR Biomedical Research Centre, Wellcome Trust Centre for Human Genetics, University of Oxford, Oxford OX3 7BN, UK; [25]Institute of Cancer Research, Cotswold Road, Sutton, Surrey SM2 5NG, UK; [26]Department of Pathology, Leiden University Medical Center, 2300 Leiden, the Netherlands; [27]Institute for Research in Biomedicine, The Barcelona Institute of Science and Technology, Barcelona, Spain; [28]Cancer Institute, University College London, 72 Huntley Street, London WC1E 6BT, UK; [29]Manchester Interdisciplinary Biocentre, University of Manchester, Manchester M1 7DN, UK; [30]Barts Cancer Institute, Barts and The London School of Medicine and Dentistry, Queen Mary University of London, London, UK; [31]Centre for Epidemiology and Biostatistics, Melbourne School of Population and Global Health, The University of Melbourne, Parkville, VIC,

(Affiliations continued on next page)

© 2022 The Author(s). This is an open access article under the CC BY license (http://creativecommons.org/licenses/by/4.0/).

David J. Adams,[14] Simone A.M. van der Biezen,[4] Michael Christie,[3,15] Mark Clendenning,[15,16] Laura E. Thomas,[17] Constantinos Deltas,[18] Aleksandar J. Dimovski,[19] Dagmara Dymerska,[20] Jan Lubinski,[20] Khalid Mahmood,[15,16] Rachel S. van der Post,[21] Mathijs Sanders,[22] Jürgen Weitz,[23] Jenny C. Taylor,[24] Clare Turnbull,[25] Lilian Vreede,[4] Tom van Wezel,[26] Celina Whalley,[1] Claudia Arnedo-Pac,[27] Giulio Caravagna,[25] William Cross,[28] Daniel Chubb,[25] Anna Frangou,[5] Andreas J. Gruber,[29] Ben Kinnersley,[25] Boris Noyvert,[1] David Church,[5] Trevor Graham,[30] Richard Houlston,[25] Nuria Lopez-Bigas,[27] Andrea Sottoriva,[28] David Wedge,[29] Genomics England Research Consortium, The CORGI Consortium, WGS500 Consortium, Mark A. Jenkins,[16,31] Roland P. Kuiper,[4,32] Andrew W. Roberts,[3,6,16,33] Jeremy P. Cheadle,[2] Marjolijn J.L. Ligtenberg,[4,21] Nicoline Hoogerbrugge,[4] Viktor H. Koelzer,[34] Andres Dacal Rivas,[35] Ingrid M. Winship,[36,37] Clara Ruiz Ponte,[38] Daniel D. Buchanan,[15,16,36] Derek G. Power,[39] Andrew Green,[40] Ian P.M. Tomlinson,[8,42,*] Julian R. Sampson,[2,42,*] Ian J. Majewski,[3,31,42] and Richarda M. de Voer[4,42]

remaining polyposis genes in order to plan appropriate tumor surveillance for affected individuals and their relatives. Here, by applying whole-genome and whole-exome sequencing (WGS and WES), we identified loss-of-protein-function (LOF) variants of the BER gene *MBD4* as the cause of an autosomal recessive syndrome of colorectal polyposis and extracolonic neoplasia.

We performed WGS or WES of constitutional DNA in a cohort of 309 individuals, from 198 apparently unrelated families, who were affected by multiple colorectal adenomas or familial CRC. For all individuals included in our study, routine diagnostic molecular genetic testing failed to detect pathogenic germline variants in known CRC and polyposis predisposing genes (detailed cohort descriptions in supplemental methods). The study received ethical approval from UK NHS Research Ethics Committees (REC numbers 06/Q1702/99 and 12/WA/0071), the Human Research Ethics Committees at the University of Melbourne (HREC #1954921) ,and the Radboudumc CMO Local Ethics Committee (#2015/2172). All participants provided written informed consent. Following WGS or WES, we prioritized the identification of coding germline variants predicted to cause LOF. This approach identified two individuals with bi-allelic frameshift variants in *MBD4*. *MBD4* encodes a BER glycosylase that repairs G:T mismatches resulting from the deamination of 5′-methylcytosine (5mC). Simplex individual D:II-1 was homozygous for a 4-bp *MBD4* deletion (GenBank: NM_003925.2: c.612_615del [p.Ser205Thrfs*9]; Figures S1A and S1E–S1H) and the other (CRDFF-292:II-3) was homozygous for an adenine duplication (GenBank: NM_003925.2: c.939dup [p.Glu314Argfs*13]; Figure S1B). Region of homozygosity analysis did not suggest consanguinity in either of the two individuals (data not shown). Both variants were exceedingly rare in gnomAD (allele frequencies 0.0000399 and 0.000653, respectively), although the c.939dup variant is one of the most common LOF variants in *MBD4* in gnomAD and was found across multiple populations. No individuals with homozygous germline LOF *MBD4* variants were found in gnomAD, the UK 100,000 Genome Project (100KGP), or the whole-genome-sequenced individuals in UK Biobank.

Next, we undertook targeted sequencing of *MBD4* in replication cohorts comprising a total of 1,611 individuals with at least ten colorectal adenomas, familial or early onset CRC, or CRC in combination with other tumors. This identified one additional, unrelated individual (CRDFF-336-1:II-1) who was homozygous for the same adenine duplication (GenBank: NM_003925.2: c.939dup; Figure S1C) and four heterozygous carriers of LOF variants in *MBD4*. While the frequency of heterozygous carriers was significantly higher than in gnomAD (4/1,611 versus 48/64,600; $p = 0.0381$, Fisher's exact), we did not confirm this enrichment in the 100KGP and UK Biobank datasets (all comparisons $p > 0.05$, Fisher's exact; Table S3).

The pedigrees of the three individuals with homozygous *MBD4* germline variants are shown in Figure 1. After genotyping of available additional family members, all were compatible with an autosomal recessive trait. Most individuals with bi-allelic LOF variants in *MBD4* developed multiple colorectal adenomas and an extracolonic neoplasm (Table 1). Simplex individual D:II-1 (Figure 1A) was found to have approximately 60 colorectal adenomas at initial colonoscopy at 36 years of age and at least 70 adenomas were identified at panproctocolectomy at 47 years of age (Figure S1H). 7 months after surgery, he was diagnosed with myelodysplastic syndrome (MDS) that

Australia; [32]Princess Máxima Center for Pediatric Oncology, 3584 Utrecht, the Netherlands; [33]University of Melbourne, Department of Medical Biology, 1G Royal Parade, Parkville, VIC 3052, Australia; [34]Department of Pathology and Molecular Pathology, University Hospital Zurich, University of Zurich, Zürich, Switzerland; [35]Servicio de Digestivo, Hospital Lucus Augusti, Instituto de Investigación Sanitaria de Santiago, Lugo, Galicia, Spain; [36]Genomic Medicine and Family Cancer Clinic, Royal Melbourne Hospital, Melbourne, VIC, Australia; [37]Department of Medicine, Melbourne Medical School, Faculty of Medicine, Dentistry and Health Sciences, University of Melbourne, Melbourne, VIC, Australia; [38]Fundación Pública Galega de Medicina Xenómica SERGAS, Grupo de Medicina Xenómica-USC, Instituto de Investigación Sanitaria de Santiago, Centro de Investigación Biomédica en Red de Enfermedades Raras, Santiago de Compostela, Galicia, Spain; [39]Department of Medical Oncology, Cork University Hospital, Cork, Ireland; [40]Department of Clinical Genetics, Children's Health Ireland, Dublin, Ireland; School of Medicine University College, Dublin, Ireland
[41]These authors contributed equally
[42]These authors contributed equally
*Correspondence: ian.tomlinson@igmm.ed.ac.uk (I.P.M.T.), sampson@cf.ac.uk (J.R.S.)
https://doi.org/10.1016/j.ajhg.2022.03.018.

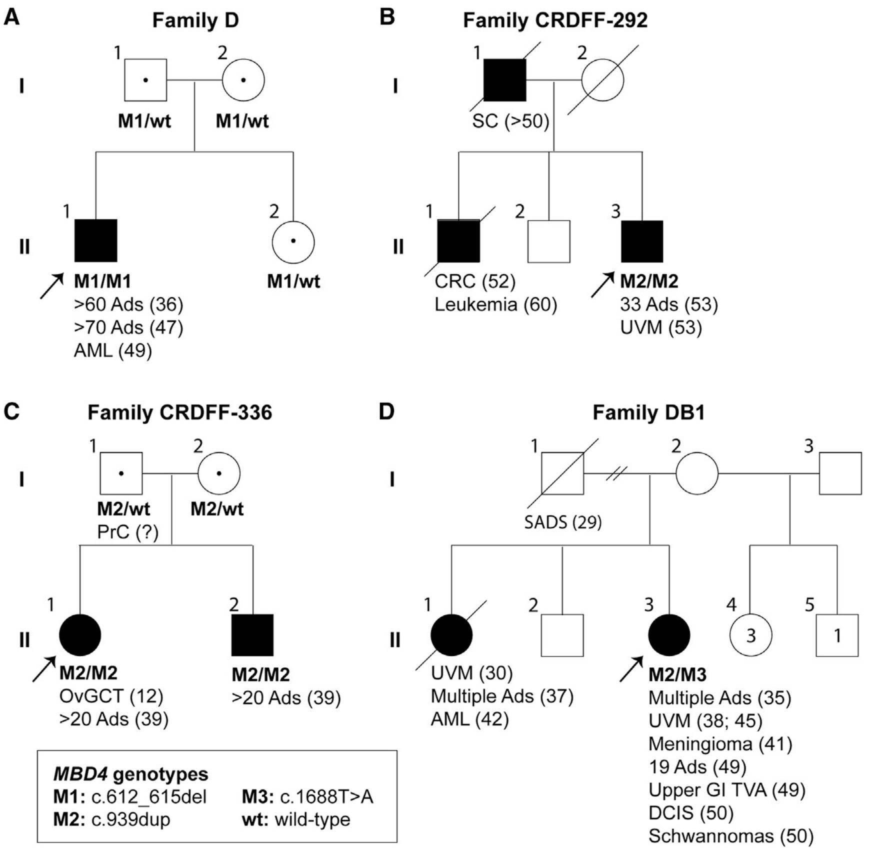

Figure 1. Pedigrees of individuals with MBD4 deficiency

(A) Pedigree of simplex individual D:II-1 with a homozygous *MBD4* loss-of-function (c.612_615del) variant. For colorectal adenomas, we show the cumulative tumor numbers from age at first presentation and screening colonoscopy to age at last contact (see also Figures S1A and S1E–S1H).
(B) Pedigree of Family CRDFF-292 with a homozygous *MBD4* loss-of-function (c.939dup) variant. For colorectal adenomas, we show the total tumor numbers identified from panproctocolectomy at age 53 (see also Figure S1B).
(C) Pedigree of Family CRDFF-336 with a homozygous *MBD4* loss-of-function (c.939dup) variant. For colorectal adenomas, we show the total tumor numbers identified from panproctocolectomy at 39 years of age (see also Figure S1C).
(D) Pedigree of Family DB1 with the bi-allelic *MBD4* loss-of-function c.939dup and c.1688T>A variants (see also Figure S1D).
Abbreviations: Ads, colorectal adenomas; AML, acute myeloid leukemia; CRC, colorectal cancer; DCIS, ductal carcinoma *in situ* of the breast; OvGCT, ovarian granulosa cell tumor; PrC, prostate cancer; SADS, sudden arrhythmia death syndrome; SC, stomach cancer; UVM, uveal melanoma; upper GI TVA, upper gastrointestinal ampullary tubulovillus adenoma; question mark, age unknown; MT, mutation; WT, wild-type. Arrows indicate index individuals. Number between brackets indicate age at diagnosis.

progressed to acute myeloid leukemia (AML) within 3 months. CRDFF-292-1 (individual II-3 in Figure 1B) had 33 colorectal adenomas at panproctocolectomy at 53 years of age and was diagnosed concurrently with a uveal melanoma. A CT scan also revealed multiple liver cysts and multiple, bilateral small renal cysts. His brother (individual II-1 in Figure 1B) had been diagnosed with colorectal cancer at 52 years of age and had died of leukemia aged 60, but no material was available for genetic testing. CRDFF-336-1 (individual II-1 in Figure 1C) had 20 colorectal adenomas at panproctocolectomy at 39 years of age and previously had surgical removal of an ovarian granulosa cell tumor at 12 years of age. Genotyping confirmed that her brother (CRDFF-336-2, individual II-2 in Figure 1C) was also homozygous for the adenine duplication (c.939dup), and colonoscopy at 39 years of age revealed approximately 20 colorectal polyps that were confirmed histologically to be adenomas with low grade dysplasia.

Two of three individuals with AML previously identified to have MBD4 deficiency were noted to have colorectal polyps, without information on their type or multiplicity.[10] We therefore obtained more comprehensive clinicopathological information on their colorectal tumors (Table 1). Individual WEHI-2 (previously WEHI-AML-2[10]) developed a total of 17 colorectal polyps over a period of 22 years from the age of 18 years. Histological assessment classified all available polyps ($n = 12$) as tubular adenomas with mild-to-moderate dysplasia, and the majority ($n = 7$) were found in the rectum (Figure S1I). A moderately differentiated adenocarcinoma was found in the ascending colon at age 40 and the individual underwent a right hemicolectomy. Individual EMC-AML-1 developed multiple colonic polyps and underwent a hemicolectomy at age 31, although no polyp counts were reported and tissue blocks were unavailable for histological re-assessment. The third individual (WEHI-AML-1[10]) did not have gastrointestinal assessment prior to her death.

We performed WES on DNA extracted from fresh-frozen or formalin-fixed paraffin-embedded (FFPE) tissue from 11 colorectal adenomas from D:II-1 and eight colorectal adenomas from WEHI-2 (Table S2). The mutation burden was increased significantly in colorectal adenomas from both individuals with MBD4 deficiency compared to previously published multi-region WES data from nine sporadic fresh-frozen adenomas[11] (Figure 2A; Table S2). The excess mutations were almost all CpG>TpG transitions (>95%) that accumulated steadily over time (Figure 2B) and were significantly more prevalent (Fisher's exact, $p = 2.9 \times 10^{-7}$) than in the sporadic colorectal

Table 1. Clinical phenotype of individuals with bi-allelic germline *MBD4* loss-of-protein-function variants

Individual	cDNA change (GenBank: NM_003925.2)	Amino acid change	M/F	Malignancies	Polyps	Benign lesions
D:II-1	c.612_615del (homozygous)	p.Ser205Thrfs*9	M	AML (49)	>130 A	N/A
CRDFF-292-1:II-3	c.939dup (homozygous)	p.Glu314Argfs*13	M	UVM (53)	33 A	liver cysts (53), bilateral small renal cysts (53)
CRDFF-336-1:II-1	c.939dup (homozygous)	p.Glu314Argfs*13	F	OvGCT (12)	>20 A	N/A
CRDFF-336-2:II-2	c.939dup (homozygous)	p.Glu314Argfs*13	M	N/A	>20 A	N/A
DB1-70:II-3	c.939dup/c.1688T>A	p.Glu314Argfs*13/ p.Leu563*	F	UVM (38, 45)	multiple A (35); 19 A (49); upper GI TVA (49)	meningioma (41); DCIS (50); schwannomas (50)
WEHI-2[10]	c.939dup/c.1562−1G>T	p.Glu314Argfs*13/ abnormal splicing	F	AML (34); CRC (40)	17 A	N/A
WEHI-AML-1[10]	c.939dup/c.1562−1G>T	p.Glu314Argfs*13/ abnormal splicing	F	AML (31)	no colonoscopy performed	N/A
EMC-AML-1[10]	c.1699_1701del (homozygous)	p.His567del	M	AML (33)	multiple A	N/A

M, male; F, female; AML, acute myeloid leukemia; UVM, uveal melanoma; OvGCT, ovarian granulosa cell tumor; CRC, colorectal cancer; A, colorectal adenomas (numbers indicate total cumulative number of colorectal polyps unless stated otherwise); upper GI TVA, upper gastrointestinal tract tubulovillus adenoma; DCIS, ductal carcinoma *in situ* of the breast; N/A not applicable. Numbers in parentheses refer to the age of diagnosis of the affected individual. Unspecified number of polyps is indicated as "multiple."

adenomas (Figure 2C). The mutation spectrum was almost exclusively attributable to COSMIC mutational signature SBS1[12] in colorectal adenomas from individuals with MBD4 deficiency, in contrast to sporadic colorectal adenomas (Figure 2D; Figures S2 and S3). This is fully consistent with a failure to repair G:T mismatches resulting from deamination of 5′-methylcytosine caused by loss of MBD4 function. Furthermore, virtually all mutated sites were methylated in normal colon (>96% of sites mutated compared to 58% of all exonic CpG sites; Figure 2E).

The driver genes mutated in MBD4-deficient adenomas were similar to those in sporadic adenomas and CRCs (Table S4). All MBD4-deficient adenomas (those that underwent WES and one additional adenoma from WEHI-2 that was targeted sequenced; see also Table S2 and the supplemental methods) harbored somatic driver mutations in *APC* with a significant enrichment of the CpG>TpG transition (GenBank: NM_000038.4: c.4348C>T) resulting in p.Arg1450*, compared with sporadic adenomas and CRCs (Fisher's exact; $p < 0.00001$; Figure 2F). MBD4-deficient adenomas harbored fewer *KRAS* mutations (three of 19 adenomas) than sporadic tumors (Fisher's exact, $p = 0.0028$) but significantly more somatic mutations in *AMER1* (MIM: 300647) (12 of 19 adenomas; Fisher's exact, $p = 0.039$) (Figure 2F). Overall, 88% of driver mutations in adenomas from individuals with MBD4 deficiency were CpG>TpG transitions compared to only 37% in sporadic adenomas (Table S4).

Incorporation of *MBD4* into diagnostic gene panels for colorectal polyposis, AML, and uveal melanoma at one of our centers has led to the identification of a further individual (DB1-70) with *MBD4*-associated neoplasia syndrome (MANS). DB1-70 (individual II-3 in Figure 1D) is compound heterozygous for the *MBD4* variants c.939dup and c.1688T>A (p.Leu563*; Figure S1D). She developed multiple adenomatous polyps in the colon at age 35, underwent a left hemicolectomy at age 39, and had 19 adenomatous polyps removed from her residual colon at age 49. DB1-70 was also diagnosed with two uveal melanomas, one at age 38 and one at age 45 years, a meningioma at age 41, a ductal carcinoma *in situ* of the breast at age 50, and a chest wall and cervical schwannoma at age 50. At age 52, she was diagnosed with liver metastases from the uveal melanoma (Figure 1D). Her sister, for whom no material was available for genetic testing, was diagnosed with a uveal melanoma at age 30 and duodenal polyps and multiple adenomatous polyps in the colon, for which a right hemicolectomy was performed, at age 37. She was diagnosed with AML at age 42 and died a year later.

Following the discovery that bi-allelic *MBD4* LOF variants predispose to AML,[10] we here show that inherited MBD4 deficiency causes a wider neoplastic syndrome including adenomatous polyposis with a colorectal phenotype similar to attenuated familial adenomatous polyposis (MIM: 175100) and to individuals with germline pathogenic variants in *MUTYH*, *NTHL1*, *POLE*, and *POLD1*. Loss of MBD4 function leads to an accumulation of somatic CpG>TpG mutations, including in well-known CRC driver genes, arising from spontaneous deamination of 5′-methylcytosine, creating a mutational signature very similar to COSMIC SBS1. We suggest the name *MBD4*-associated neoplasia syndrome (MANS) for this condition.

To date, colorectal polyposis, MDS/AML, and uveal melanoma appear to be the most common clinical manifestations of MANS. Thus far, to our knowledge, all individuals that have had a colonoscopy have had multiple colorectal polyps early in life, and most have experienced MDS/AML. Identification of individuals with bi-allelic *MBD4*

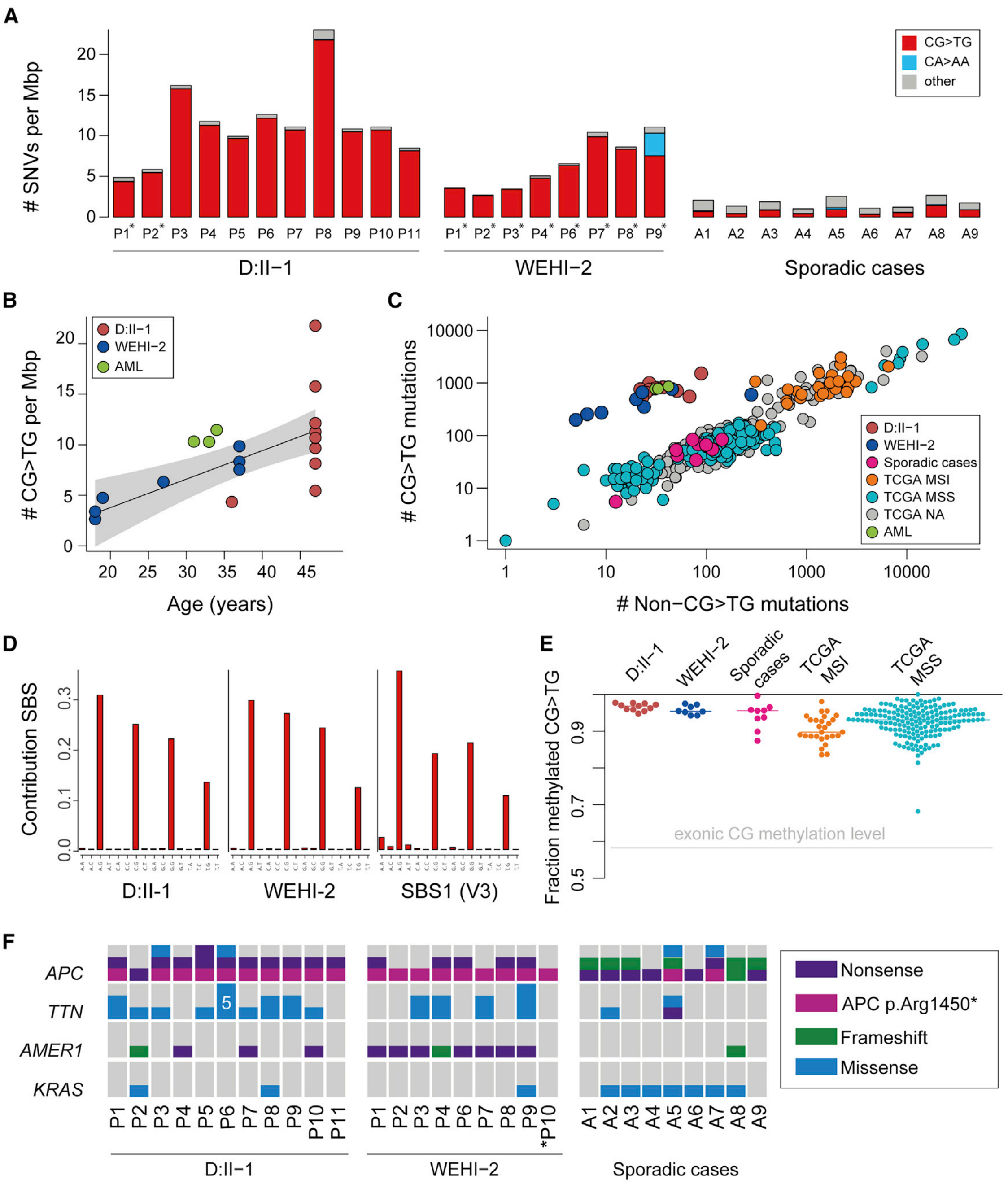

Figure 2. Somatic mutation burden and analysis of polyps of individuals with MBD4 deficiency

(A) Somatic mutation rate for each polyp, formalin-fixed and paraffin-embedded samples indicated with asterisks (*). The color of the bars represents mutations in different sequence contexts; red shows CG>TG mutations, blue shows CA>AA mutations (primarily detected in WEHI-2 P9), and gray represents other base contexts. The median value is presented for samples that had multi-region sequencing. Median mutation burden/Mb in fresh frozen adenomas of D:II-1 was 11.1 [range 8.5–23.3] compared to 1.8 in a set of nine fresh frozen sporadic adenomas [range 1.0–3.1] (see also Figures S1H–S1I for representative HE slides).

(B) The number of somatic CG>TG mutations detected in WES data is plotted as function of age. The linear fit is shown, together with 95% confidence intervals (gray shading).

(C) We assessed the contribution of deamination of 5mC to MBD4-deficient samples by comparing the number of CG>TG mutations to all other single-nucleotide mutations. The plot compares MBD4-deficient polyps and AMLs[12] to sporadic polyps, and to colon and rectal cancers from The Cancer Genome Atlas (TCGA). MSI, microsatellite instability; MSI-H, MSI-high; MSS, microsatellite stable ("MSS" includes both MSS and MSI-low samples); TCGA NA, no MSI data available.

(D) Extracted *de novo* signature SBS1^{MBD4} C>T panel from all polyps from D:II-1(left) and polyps P1–P8 of WEHI-2 (middle) and the C>T panel from COSMIC SBS1-v3 (right).

(E) Fraction of mutated CpG sites that are methylated in normal sigmoid colon (beta value > 0.5 in WGBS data from the Roadmap Epigenomics Consortium[11]). Each point summarizes WES results from a sample and includes all sites with sufficient coverage in

(legend continued on next page)

pathogenic variants may inform their clinical management and that of their families. The identification and follow up of additional individuals will help to define the magnitude of cancer risks in MANS. In the interim, we propose colonoscopies every 2 years from age 18–20, or the date of diagnosis, a regimen often used for other BER-related polyposis syndromes.[13,14] At least one of the AMLs in our study developed from MDS, and we have observed clonal hematopoiesis in others.[10] We suggest regular follow up full blood counts for individuals with MANS if their initial presentation is with adenomatous polyposis. If the individual presents with AML, then we suggest genetic testing for any family member being considered as a haematopoietic stem cell donor, in keeping with current expert recommendations for managing inherited predisposition to myeloid malignancy.[15,16] Given that heterozygous LOF *MBD4* variant carriers appear to be susceptible to uveal melanoma[17–19] and our identification of uveal melanoma in three of eight individuals with MANS suggests annual ophthalmological surveillance may also be appropriate.[20] The occurrence of a rare juvenile ovarian granulosa cell tumor in one of four females and schwannomas in another individual reported here is noteworthy and the spectrum of MBD4-deficiency-associated cancers may widen as further individuals with MANS are identified. In contrast to findings with uveal melanoma where heterozygotes for *MBD4* LOF variants appear to be at a 4- to 20-fold increased risk, our limited data show no convincing evidence for a comparable effect on the relative risk of developing polyposis and/or CRC. We cannot rule out the possibility that individuals heterozygous for an *MBD4* LOF variant have a small increased risk of CRC and/or polyposis,[21] but at present, no colonoscopy surveillance beyond population screening or local guidelines based on familial history for CRC is recommended. Additionally, although it has been suggested that variable expression of MBD4 contributes to differences in DNA repair capacity,[22] further investigation is required to determine whether this contributes to modify disease risk.

In conclusion, constitutional deficiency of MBD4 causes a rare genetic syndrome, MANS, that is characterized by the development of adenomatous polyposis and predisposition to AML. MBD4 deficiency results in an elevated mutation burden with a mutation spectrum very similar to COSMIC mutational signature SBS1. A high mutational burden is associated with a good prognosis in CRC, and we speculate that MANS CRCs may respond to immune checkpoint inhibitors, as has been reported in MBD4-deficient uveal melanomas.[17,18] It is possible that such a strategy could also be used to treat other neoplasia in MANS. In the short term, genetic testing for MANS could be implemented readily by incorporating *MBD4* into existing gene panels used in diagnostic testing for adenomatous polyposis, CRC, early-onset AML, and uveal melanoma.

Data and code availability

The WES data from WEHI-2 and D:II-1 datasets generated during this study are available at EGA (EGA: S00001004842 and EGA: S00001005063, respectively) after completion of a data transfer agreement. The WES data from CRDFF-292 supporting the current study have not been deposited in a public repository because of informed consent restrictions but are available from the corresponding author on request. WES/WGS data for ACCFR in the paper is available from the Colon Cancer Family Registry (https://www.coloncfr.org). Somatic variants in selected driver genes are available in Table S4. Somatic variant calls are available from the corresponding author on request.

Supplemental information

Supplemental information can be found online at https://doi.org/10.1016/j.ajhg.2022.03.018.

Consortia

The members of Genomics England Research Consortium are John Ambrose, Prabhu Arumugam, Marta Bleda, Freya Boardman-Pretty, Christopher Boustred, Helen Brittain, Mark Caulfield, Georgia Chan, Tom Fowler, Adam Giess, Angela Hamblin, Shirley Henderson, Tim Hubbard, Rob Jackson, Louise Jones, Dalia Kasperaviciute, Melis Kayikci, Athanasios Kousathanas, Lea Lahnstein, Sarah Leigh, Ivone Leong, Javier Lopez, Fiona Maleady-Crowe, Meriel McEntagart, Federico Minneci, Loukas Moutsianas, Michael Mueller, Nirupa Murugaesu, Anna Need, Peter O'Donovan, Chris Odhams, Christine Patch, Daniel Perez-Gil, Mariana Pereira, John Pullinger, Tahrima Rahim, Augusto Rendon, Tim Rogers, Kevin Savage, Kushmita Sawant, Richard Scott, Afshan Siddiq, Alexander Sieghart, Samuel Smith, Alona Sosinsky, Alexander Stuckey, Mélanie Tanguy, Ana Tavares, Ellen Thomas, Simon Thompson, Arianna Tucci, Matthew Welland, Eleanor Williams, Katarzyna Witkowska, and Suzanne Wood.
The members of the CORGI Consortium are Kai Ren Ong, Andrew Beggs, Alan Donaldson, Carole Brewer, Jayantha Arnold, Munaza Ahmed, Louise Izatt, Andrew Latchford, Dorothy Halliday, Peter Risby, Paul Brennan, Alison Kraus, Julian Barwell, Lynn Greenhalgh, D. Gareth Evans, Kate Green, Timothy Simmons, Rachel Harrison, Ragunath, Brian Davidson, Zoe Kemp, Helen Hanson, Katie Snape, Anneke Lucassen, Kevin J. Monahan, and Patrick Morrison.
The members of WGS500 Consortium are Peter Donnelly, John Bell, David Bentley, Gil McVean, Peter Ratcliffe, Jenny Taylor, Andrew Wilkie, John Broxholme, David Buck, Jean-Baptiste Cazier, Richard Cornall, Lorna Gregory, Julian Knight, Gerton Lunter, Ian Tomlinson, Andrew Wilkie, Christopher Allan, Moustafa Attar, Angie Green, Lorna Gregory, Sean Humphray, Zoya Kingsbury,

WGBS (n = 177–1,507 CG>TG mutations) and the median value is shown with a horizontal line. The gray line shows the fraction of methylated CG sites across all exons.
(F) Oncoprint of driver gene mutation analysis of genes significantly different mutated compared to sporadic adenomas. For each polyp, the number and type of somatic mutation is shown. *Polyp P10 from WEHI-2 was sequenced with a targeted panel (see also Table S5).

Sarah Lamble, Lorne Lonie, Alistair Pagnamenta, Paolo Piazza, Amy Trebes, John Broxholme, Richard Copley, Simon Fiddy, Russell Grocock, Edouard Hatton, Chris Holmes, Linda Hughes, Peter Humburg, Alexander Kanapin, Stefano Lise, Hilary Martin, Lisa Murray, Davis McCarthy, Andy Rimmer, Natasha Sahgal, Ben Wright, and Chris Yau.

Acknowledgments

This study was funded by the Dutch Cancer Society (KUN2015-7740; 12174/2019-1), the Sacha Swarttouw-Hijmans Foundation, Cancer Research UK C6199, Bowel Cancer West David Darke grant, the EU ERC (EVOCAN), the National Health and Medical Research Council of Australia (project 1145912, program 1113577, investigator 1174902) and the Cancer Council Victoria (1181108), with fellowship support from the Victorian Cancer Agency (I.J.M., MCRF15018), the Alfred Felton Bequest (I.J.M.) and the Leukaemia Foundation of Australia (Bill Long Charitable Trust PhD Clinical Scholarship to E.C.), and Bowel Cancer UK (CP 18PG0010). H.W. is supported by a Ser Cymru II Precision Medicine Fellowship award. Research was also supported by the Australian Cancer Research Foundation, Victorian State Government Operational Infrastructure Support, and Australian Government NHMRC IRIISS. D.D.B. is supported by an NHMRC R.D. Wright Career Development Fellowship (GNT1125268) and NHMRC Emerging Leadership Fellowship (GNT1194896). M.A.J. is supported by NHMRC Leadership Fellowship. The ACCFR is supported by funding from the National Cancer Institute (NCI), National Institutes of Health (NIH) (award U01 CA167551). The research was also supported by the National Institute for Health Research (NIHR) Oxford Biomedical Research Center based at Oxford University Hospitals NHS Trust and University of Oxford. J.C.T. discloses that the views expressed are those of the author(s) and not necessarily those of the NHS, the NIHR, or the Department of Health. This work received networking support by the Cooperation in Science and Technology Action CA17118, supported by the European Cooperation in Science and Technology. Further acknowledgments are described in the supplemental information.

Author contributions

Study supervision: C.P., J.R.S., I.P.M.T., I.J.M., and R.M.d.V. Analysis and drafting: C.P., E.C., J.E.G., S.G., C.F., H.W., J.R.S., I.P.M.T., I.J.M., and R.M.d.V. Data support: E.A.M.J., H.C., L.C., E.A.B., N.L., A.B., R.T., J.P., K.S., L.M., H.T., D.G., F.F., Y.G., D.J.A., S.A.M.v.d.B., M. Christie, M. Clendenning, L.E.T., C.D., J.A.D., D.D., J.L., K.M., R.S.v.d.P., M.S., J.W., J.C.T., C.T., L.V., T.v.W., C.W., C.A., G.C., W.C., D.C., A.F., A.G., B.K., B.N., D.C., T.G., R.H., N.L.B., A.S., D.W., M.A.J., R.P.K., A.W.R., J.P.C., M.J.L.L., N.H., V.H.K., A.D.R., I.M.W., C.R.P., D.D.D., D.G.P., and A.G. Critical revision: all authors. Shared last authors: J.R.S., I.P.M.T., I.J.M., and R.M.d.V.

Declaration of interests

The authors declare no competing interests.

Received: January 21, 2022
Accepted: March 30, 2022
Published: April 22, 2022

Web resources

Online Mendelian Inheritance in Man, http://www.omim.org

References

1. Palles, C., Cazier, J.B., Howarth, K.M., Domingo, E., Jones, A.M., Broderick, P., Kemp, Z., Spain, S.L., Guarino, E., Salguero, I., et al.; The WGS500 Consortium (2013). Germline mutations affecting the proofreading domains of POLE and POLD1 predispose to colorectal adenomas and carcinomas. Nat. Genet. *45*, 136–144. https://doi.org/10.1038/ng.2503.
2. Will, O., Carvajal-Carmona, L.G., Gorman, P., Howarth, K.M., Jones, A.M., Polanco-Echeverry, G.M., Chinaleong, J.A., Gunther, T., Silver, A., Clark, S.K., and Tomlinson, I. (2007). Homozygous PMS2 deletion causes a severe colorectal cancer and multiple adenoma phenotype without extraintestinal cancer. Gastroenterology *132*, 527–530. https://doi.org/10.1053/j.gastro.2006.11.043.
3. Wimmer, K., Kratz, C.P., Vasen, H.F.A., Caron, O., Colas, C., Entz-Werle, N., Gerdes, A.M., Goldberg, Y., Ilencikova, D., Muleris, M., et al.; on behalf of the EU-Consortium Care for CMMRD C4CMMRD (2014). Diagnostic criteria for constitutional mismatch repair deficiency syndrome: suggestions of the European consortium 'Care for CMMRD' (C4CMMRD). J. Med. Genet. *51*, 355–365. https://doi.org/10.1136/jmedgenet-2014-102284.
4. Weren, R.D.A., Ligtenberg, M.J.L., Kets, C.M., de Voer, R.M., Verwiel, E.T.P., Spruijt, L., van Zelst-Stams, W.A.G., Jongmans, M.C., Gilissen, C., Hehir-Kwa, J.Y., et al. (2015). A germline homozygous mutation in the base-excision repair gene NTHL1 causes adenomatous polyposis and colorectal cancer. Nat. Genet. *47*, 668–671. https://doi.org/10.1038/ng.3287.
5. Al-Tassan, N., Chmiel, N.H., Maynard, J., Fleming, N., Livingston, A.L., Williams, G.T., Hodges, A.K., Davies, D.R., David, S.S., Sampson, J.R., et al. (2002). Inherited variants of MYH associated with somatic GC–>T: A mutations in colorectal tumors. Nat. Genet. *30*, 227–232.
6. Drost, J., van Boxtel, R., Blokzijl, F., Mizutani, T., Sasaki, N., Sasselli, V., de Ligt, J., Behjati, S., Grolleman, J.E., van Wezel, T., et al. (2017). Use of CRISPR-modified human stem cell organoids to study the origin of mutational signatures in cancer. Science *358*, 234–238. https://doi.org/10.1126/science.aao3130.
7. Grolleman, J.E., de Voer, R.M., Elsayed, F.A., Nielsen, M., Weren, R.D., Palles, C., Ligtenberg, M.J., Vos, J.R., ten Broeke, S.W., de Miranda, N.F., et al. (2019). Mutational signature analysis reveals NTHL1 deficiency to cause a multi-tumor phenotype. Cancer Cell *35*, 256–266.e5. https://doi.org/10.1016/j.ccell.2018.12.011.
8. Pilati, C., Shinde, J., Alexandrov, L.B., Assie, G., Andre, T., Helias Rodzewicz, Z., Ducoudray, R., Le Corre, D., Zucman Rossi, J., Emile, J., et al. (2017). Mutational signature analysis identifies MUTYH deficiency in colorectal cancers and adrenocortical carcinomas. J. Pathol. *242*, 10–15. https://doi.org/10.1002/path.4880.
9. Viel, A., Bruselles, A., Meccia, E., Fornasarig, M., Quaia, M., Canzonieri, V., Policicchio, E., Urso, E.D., Agostini, M., Genuardi, M., et al. (2017). A specific mutational signature associated with DNA 8-oxoguanine persistence in MUTYH-defective colorectal cancer. EBioMedicine *20*, 39–49. https://doi.org/10.1016/j.ebiom.2017.04.022.

10. Sanders, M.A., Chew, E., Flensburg, C., Zeilemaker, A., Miller, S.E., al Hinai, A.S., Bajel, A., Luiken, B., Rijken, M., Mclennan, T., et al. (2018). MBD4 guards against methylation damage and germ line deficiency predisposes to clonal hematopoiesis and early-onset AML. Blood *132*, 1526–1534. https://doi.org/10.1182/blood-2018-05-852566.
11. Cross, W., Kovac, M., Mustonen, V., Temko, D., Davis, H., Baker, A.M., Biswas, S., Arnold, R., Chegwidden, L., Gatenbee, C., et al. (2018). The evolutionary landscape of colorectal tumorigenesis. Nat. Ecol. Evol. *2*, 1661–1672. https://doi.org/10.1038/s41559-018-0642-z.
12. John, G.T., Sondka, Z., Beare, D.M., Bindal, N., Boutselakis, H., Cole, C.G., Creatore, C., Dawson, E., Fish, P., Harsha, B., et al. (2019). COSMIC: the catalogue of somatic mutations in cancer. Cancer. Nucleic Acids Res. *47*, D941–D947. https://doi.org/10.1093/nar/gky1015.
13. Nielsen, M., Infante, E., and Brand, R. (2012). MUTYH Polyposis (GeneReviews).
14. Kuiper, R.P., Nielsen, M., De Voer, R.M., and Hoogerbrugge, N. (2020). NTHL1 tumor syndrome. Seattle (WA): university of Washington (GeneReviews).
15. Godley, L.A., and Shimamura, A. (2017). Genetic predisposition to hematologic malignancies: management and surveillance. Blood *130*, 424–432. https://doi.org/10.1182/blood-2017-02-735290.
16. University of Chicago Hematopoietic Malignancies Cancer Risk T. (2016). How I diagnose and manage individuals at risk for inherited myeloid malignancies. Blood *128*, 1800–1813. https://doi.org/10.1182/blood-2016-05-670240.
17. Johansson, P.A., Stark, A., Palmer, J.M., Bigby, K., Brooks, K., Rolfe, O., Pritchard, A.L., Whitehead, K., Warrier, S., Glasson, W., and Hayward, N.K. (2019). Prolonged stable disease in a uveal melanoma patient with germline MBD4 nonsense mutation treated with pembrolizumab and ipilimumab. Immunogenetics *71*, 433–436. https://doi.org/10.1007/s00251-019-01108-x.
18. Rodrigues, M., Mobuchon, L., Houy, A., Fievet, A., Gardrat, S., Barnhill, R.L., Popova, T., Servois, V., Rampanou, A., Mouton, A., et al. (2018). Outlier response to anti-PD1 in uveal melanoma reveals germline MBD4 mutations in hypermutated tumors. Nat. Commun. *9*, 1866. https://doi.org/10.1038/s41467-018-04322-5.
19. Derrien, A.C., Rodrigues, M., Eeckhoutte, A., Dayot, S., Houy, A., Mobuchon, L., Gardrat, S., Lequin, D., Ballet, S., Pierron, G., et al. (2021). Germline MBD4 mutations and predisposition to uveal melanoma. J. Natl. Cancer Inst. *113*, 80–87. https://doi.org/10.1093/jnci/djaa047.
20. Chau, C., van Doorn, R., van Poppelen, N.M., van der Stoep, N., Mensenkamp, A.R., Sijmons, R.H., van Paassen, B.W., van den Ouweland, A.M.W., Naus, N.C., van der Hout, A.H., et al. (2019). Families with BAP1-tumor predisposition syndrome in The Netherlands: path to identification and a proposal for genetic screening guidelines. Cancers *11*, 1114. https://doi.org/10.3390/cancers11081114.
21. Tanakaya, K., Kumamoto, K., Tada, Y., Eguchi, H., Ishibashi, K., Idani, H., Tachikawa, T., Akagi, K., Okazaki, Y., and Ishida, H. (2019). A germline MBD4 mutation was identified in a patient with colorectal oligopolyposis and early-onset cancer: A case report. Case Rep. Oncol Rep *42*, 1133–1140. https://doi.org/10.3892/or.2019.7239.
22. ICGC/TCGA Pan-Cancer Analysis of Whole Genomes Consortium (2020). Pan-cancer analysis of whole genomes. Nature *578*, 82–93. https://doi.org/10.1038/s41586-020-1969-6.

The American Journal of Human Genetics, Volume *109*

Supplemental information

Germline MBD4 deficiency causes a multi-tumor predisposition syndrome

Claire Palles, Hannah D. West, Edward Chew, Sara Galavotti, Christoffer Flensburg, Judith E. Grolleman, Erik A.M. Jansen, Helen Curley, Laura Chegwidden, Edward H. Arbe-Barnes, Nicola Lander, Rebekah Truscott, Judith Pagan, Ashish Bajel, Kitty Sherwood, Lynn Martin, Huw Thomas, Demetra Georgiou, Florentia Fostira, Yael Goldberg, David J. Adams, Simone A.M. van der Biezen, Michael Christie, Mark Clendenning, Laura E. Thomas, Constantinos Deltas, Aleksandar J. Dimovski, Dagmara Dymerska, Jan Lubinski, Khalid Mahmood, Rachel S. van der Post, Mathijs Sanders, Jürgen Weitz, Jenny C. Taylor, Clare Turnbull, Lilian Vreede, Tom van Wezel, Celina Whalley, Claudia Arnedo-Pac, Giulio Caravagna, William Cross, Daniel Chubb, Anna Frangou, Andreas J. Gruber, Ben Kinnersley, Boris Noyvert, David Church, Trevor Graham, Richard Houlston, Nuria Lopez-Bigas, Andrea Sottoriva, David Wedge, Genomics England Research Consortium, The CORGI Consortium, WGS500 Consortium, Mark A. Jenkins, Roland P. Kuiper, Andrew W. Roberts, Jeremy P. Cheadle, Marjolijn J.L. Ligtenberg, Nicoline Hoogerbrugge, Viktor H. Koelzer, Andres Dacal Rivas, Ingrid M. Winship, Clara Ruiz Ponte, Daniel D. Buchanan, Derek G. Power, Andrew Green, Ian P.M. Tomlinson, Julian R. Sampson, Ian J. Majewski, and Richarda M. de Voer

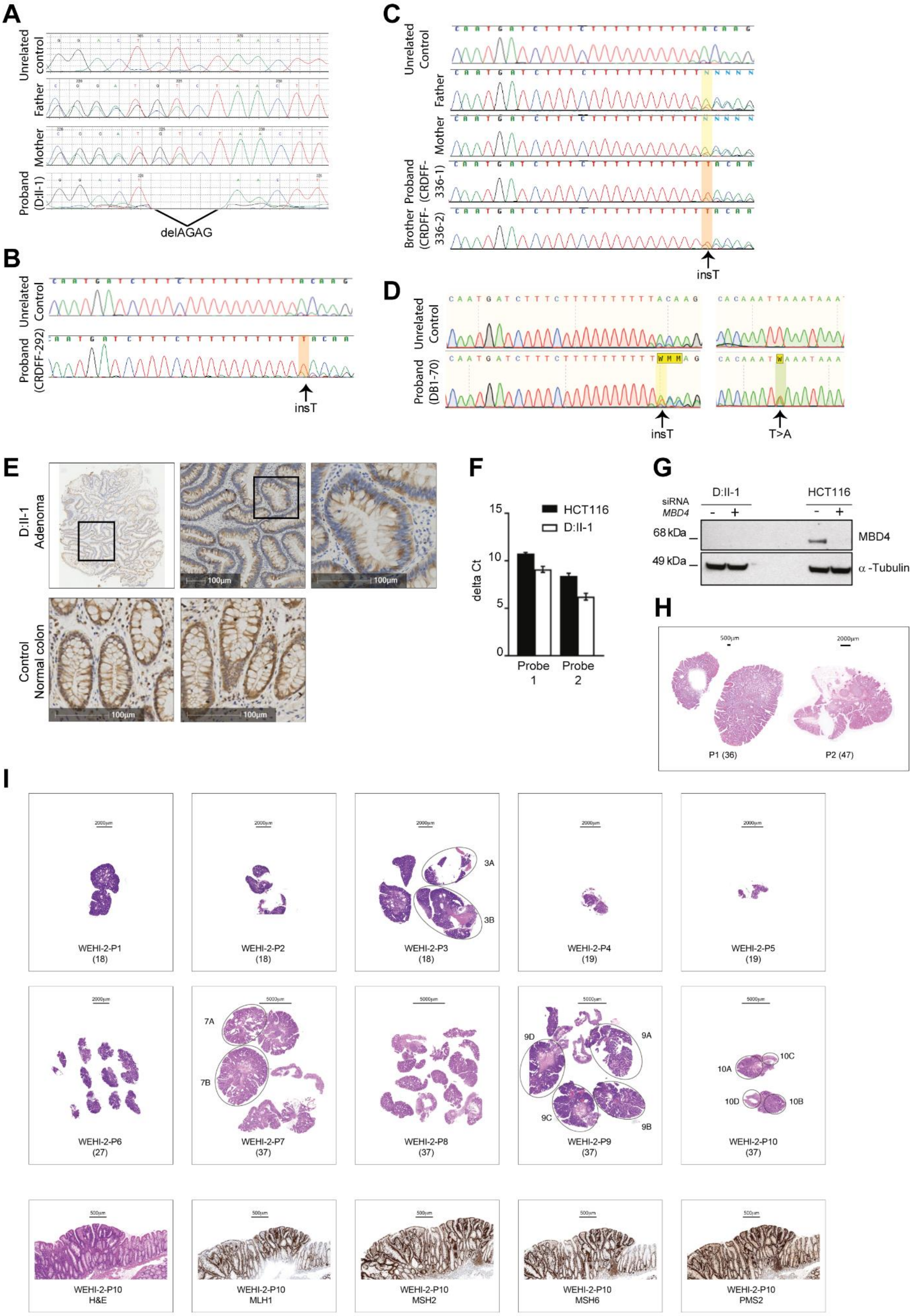

Figure S1: Sanger validations and *MBD4* expression in lymphoblastoid cells and histology of polyps from MBD4-deficient individuals. A) Sanger validations on genomic

DNA of the homozygous MBD4 loss-of-function (c.612_615del) variant in D:II-1 and parents. **B)** Sanger validations on genomic DNA (antisense strand) of the homozygous *MBD4* loss-of-function (c.939dup) variant in CRDFF-292 and unrelated control. Highlighted in orange the homozygous thymine insertion in CRDFF-292. **C)** Sanger validations on genomic DNA (antisense strand) of the homozygous *MBD4* loss-of-function (c.939dup) variant in CRDFF-336-1, parents, sibling and unrelated control. Highlighted in yellow the heterozygous thymine insertion in the parents of CRDFF-336-1. Highlighted in orange the homozygous thymine insertion in CRDFF-336-1 and her sibling CRDFF-336-2. **D)** Sanger validations on genomic DNA (antisense strand) of the heterozygous *MBD4* loss-of-function variants c.939dup (left) and c.1688T>A (right) variant in DB1-70 and unrelated control. Highlighted in yellow and green are the heterozygous thymine insertion and the heterozygous T>A change in DB1-70, respectively. **E)** Representative MBD4 IHC of an adenoma from simplex case D:II-1 (upper panels) and of a normal colon with wild type MBD4 (lower panels) stained with anti-MBD4 antibody. The D:II-1 (upper panels) show a tubular adenoma with low grade dysplasia showing typical nuclear changes (pencil shaped nuclei, crowding and pseudostrastification). **F)** RNA expression analysis showed stable expression of MBD4 as determined using two Taqman probes targeting MBD4 RNA (probe1=hS01023548; probe 2=HS00187498). Ct averages were 29.1 and 29.3 for HCT116 and D:II-1 respectively, using probe_1 and 26.7 and 26.4 for HCT116 and D:II-1 respectively, using probe_2. Bars are plotted as average of triplicate experiments with standard deviation. **G)** Western blot analysis of MBD4 expression in lymphoblastoid cells from simplex case D:Il-1 (-) and treated with an siRNA targeting MBD4 (+). The colorectal cancer cell line HCT116 was used a positive control. Two central lanes were left empty. Anti-alpha-Tubulin was used as loading control. **H)** Haematoxylin and eosin staining of polyps from D:II-1 with age in brackets. **I)** Haematoxylin and eosin staining and immunohistochemistry in polyps from WEHI-2. Rows 1 & 2: Haematoxylin and eosin (H&E) staining of sections from 10 polyps excised from WEHI-2, with age in years in brackets. Polyps P3, P7, P9 and P10 had multi-regions independently sequenced these regions are indicated by circles. Size bars represent 2000uM (P1 to P6) or 5000um (P7 to P10). Row 3: Immunohistochemistry staining of WEHI-2 P10 showing proficiency in mismatch repair proteins (MLH1, MSH2, MSH6 and PMS2). An H&E slide is shown for comparison. Size bars represent 500uM.

A

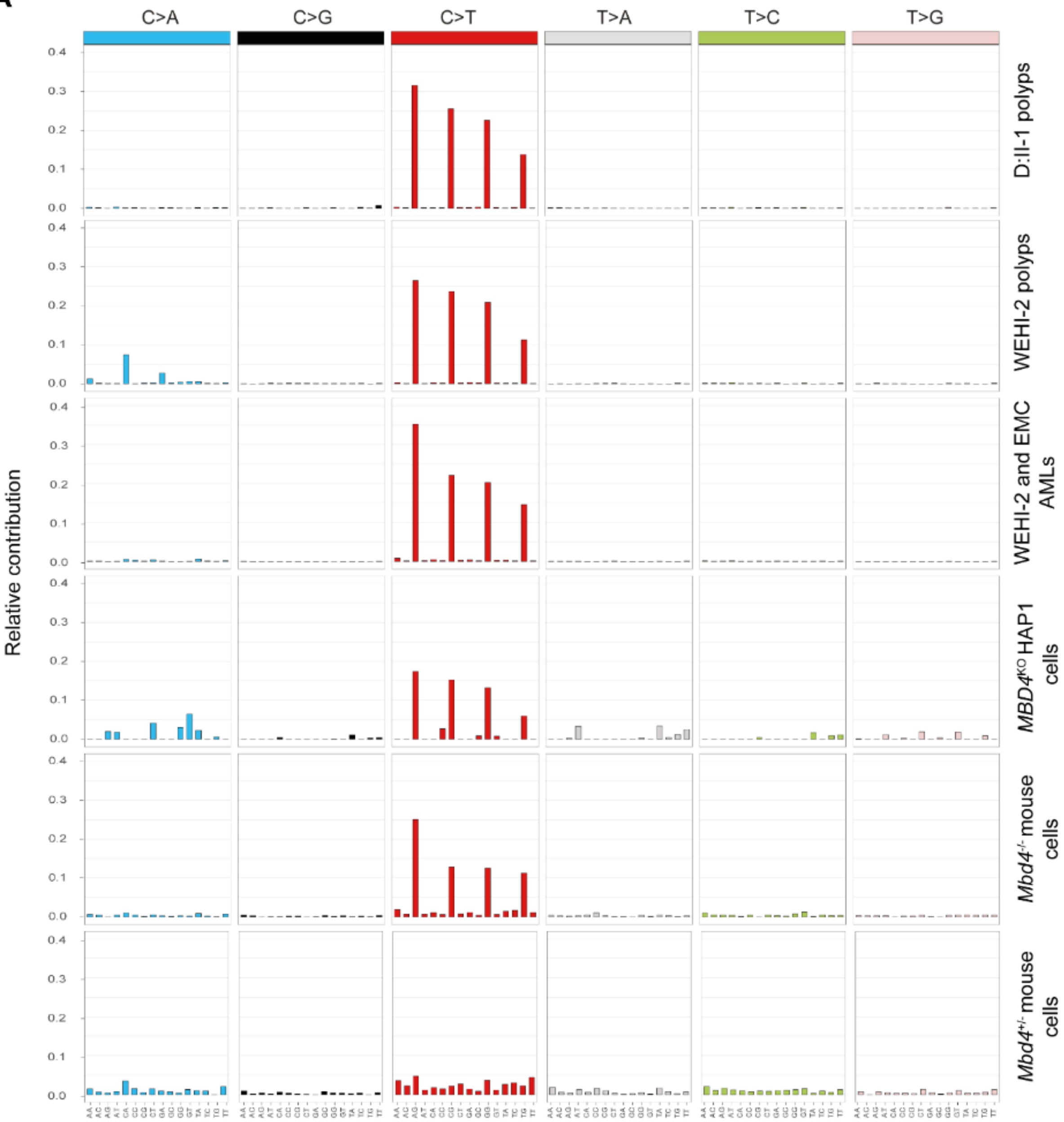

B

Mutational profiles	D:II-1	WEHI-2
SBS1-v3 (COSMIC)	0.9836	0.9602
WEHI and EMC AMLs	0.9928	0.9708
MBD4KO HAP1 cells	0.9683	0.9466
MBD4 -/- mouse cells	0.9193	0.9094
MBD4 +/- mouse cells	0.4867	0.5262

Figure S2: Mutation profiles and cosine similarity of observed mutations in various samples. A) Combined mutation profiles in the 96-mutation spectrum plot for each of the samples indicated. AML data originates from Sanders et al.[1] **B)** Cosine similarity scores indicate the closeness of the mutation profile of D:II-1 and WEHI-2 with the various mutations profiles observed in the other sequenced samples.

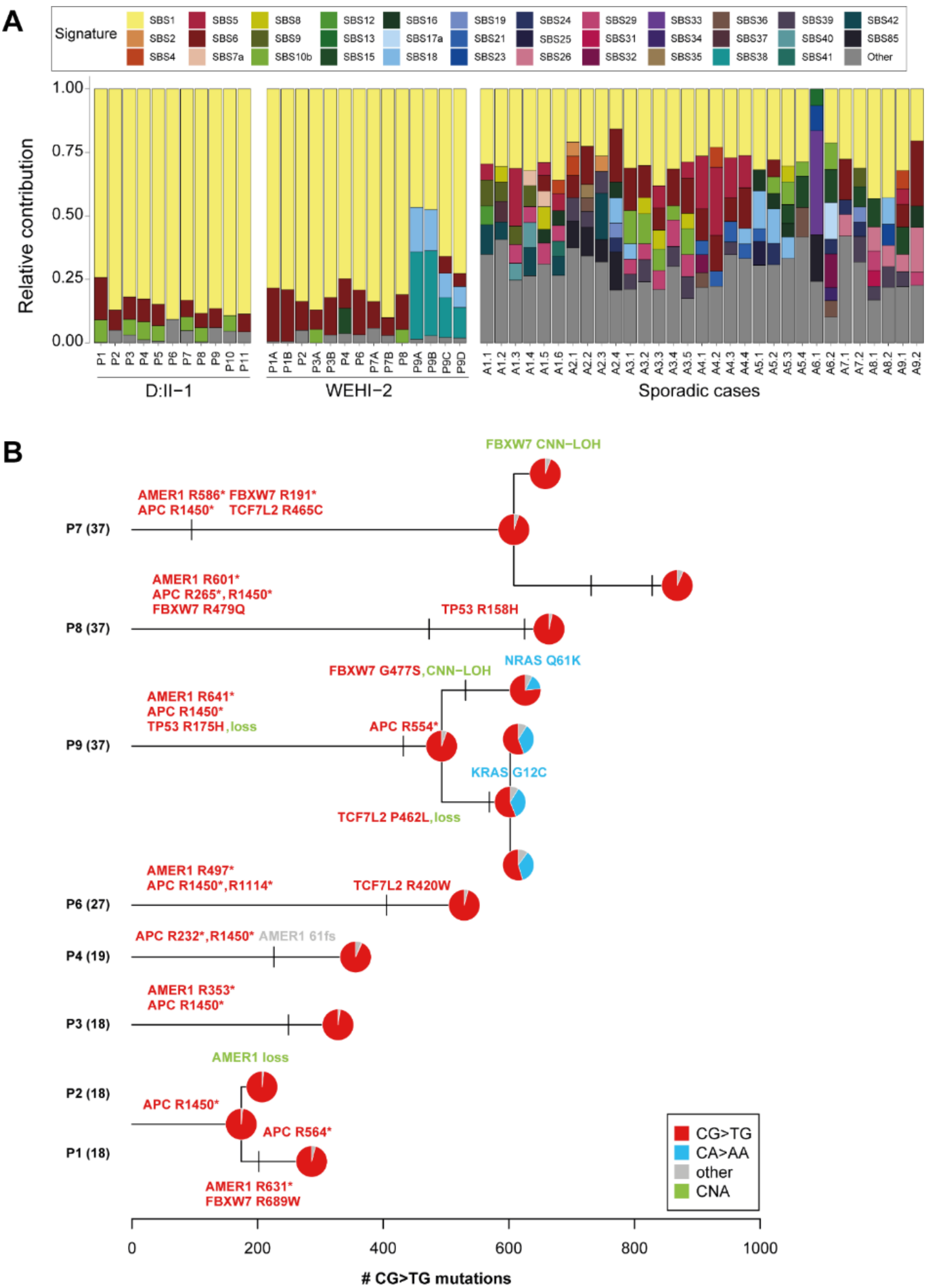

Figure S3: Mutational signature refitting of polyps of MBD4 deficient individuals and clonal evolution of polyps in WEHI-2. A) The estimated relative contribution of all known COSMIC-v3 mutational signatures to mutations in whole-exome sequenced adenomas from two MBD4-deficient individuals and nine adenomas from sporadic origin. For WEHI-2 and the sporadic polyps multi-regions were sequenced. For WEHI-2 multi-region sequenced polyps

are indicated by P1A or P1B, ect., and for the sporadic polyps this is indicated by by A1.1 and A1.2, ect. (see also Supplemental Table 2). Signatures with a contribution of less than 5% were merged into “other”. **B)** Clonal evolution of polyps in WEHI-2. These trees represents the development of clones within each polyp, with vertical bars or branches representing subclones. Note that P1 and P2 share a common precursor; labels are placed adjacent to the dominant clone in each polyp. The x-axis shows the number of somatic CG>TG mutations in each clone. Timing for key driver mutations is shown with earlier mutations on the left and the colour reflecting the type of mutation, either CG>TG (red), CA>AA (blue), copy number (green) or other (grey). All four physically distinct regions of WEHI-2 had a lower percentage of CpG>TpG transitions (55-77%) than the other adenomas (see also panel A) and a substantial proportion of mutations were CA>AA transversions (Figure 2C). The different regions were clonally related and, using CpG>TpG transitions as a molecular clock, we found that this shift in mutational profile likely coincided with treatment for AML.

Table S1: Patient cohort inclusion and results of the targeted *MBD4* screening

**Cohort**	**# samples**	**Selection Criteria**	**Genes tested negative**	**Loss of function germline variants in *MBD4***
Skopje, Macedonia	12	Polyposis, recessive inheritance	MMR genes, *APC*, *TP53*, *MUTYH*, *POLE*, *POLD1*	none
Nijmegen, the Netherlands	147	Polyposis or familial CRC	*APC*, *MUTYH*	none
Leiden, the Netherlands	149	Polyposis or familial CRC	*APC*, *MUTYH*	none
Dresden, Germany	100	Polyposis or familial CRC	*APC*, *MUTYH*	none
Oxford, United Kingdom	275	Polyposis	*APC*, *MUTYH*	c.1437G>A; heterozygous (n = 1)<br>c.1636C>T; heterozygous (*n* = 1)
Szczecin, Poland	144	Familial CRC	*POLE*, *POLD1*, MMR genes*	none
Santiago de Compostela, Spain	35	Polyposis or familial CRC	*APC*, *MUTYH* (partly), *POLE*, *POLD1, BMPR1A, SMAD4, PTEN*	c.1562-1G>T; heterozygous (*n* = 1)
ParelBED, the Netherlands	600	Polyposis with or without CRC, and/or CRC in combination with (a) different tumour(s)	No disease causing mutation found (tested negative or not tested)	c.1410A>C; heterozygous (*n* = 1)
CORGI collaborator, Greece	28	polyposis (10-100 polyps) with or without CRC	*APC, MUTYH, NTHL1 (NTHL1 tested in most samples and negative in all tested)*	none
CORGI collaborator, Cyprus	2	polyposis	unknown	none
CORGI collaborator, Australia	1	polyposis	unknown	none
Cardiff, England	118	polyposis	*APC, MUTYH*	c.939dup; homozygous (*n* = 1)
**Screening Total**	1,611			

MMR genes: MLH1, MSH2, MSH6, and PMS2. *Most patients were tested for these genes.

Table S3: Enrichment analysis of *MBD4* loss of protein function variants in individuals with polyposis and/or CRC and/or AML/MDS

Cohort (*n*)	Cases (*n*)	Controls (*n*)	OR	95% CI	*P*-value
**Replication study**					
polyposis and/or CRC (1,611)	4	na	0.3	0.11-1.14	0.038
gnomAD (64,600)	na	48	-	-	-
**UK Biobank**					
CRC (2,357)*	2	na	1.02	0.28-8.48	1
polyposis (5,116)*	1	na	4.42	0.78-175.70	0.138
AML (221)*	1	na	0.19	0.03-7.59	0.175
MDS (104)*	1	na	0.09	0.02-3.57	0.086
others (193,255)	na	167	-	-	-
**100KGP**					
CRC (2,438)#	4	na	0.74	0.25-2.98	0.542
others (17,243)	na	21	-	-	-

* Fisher's exact test compared with others in UK Biobank
# Fisher's exact test compared with others in 100KGP
Abbreviations: 100KGP: 100,000 genomes project; OR: odds ratio; CI: confidence interval; AML: acute myeloid leukaemia; MDS: myelodysplastic syndrome

Supplementary Methods:

Study cohorts and whole-genome and -exome sequencing for germline variants

i) Individuals with multiple colorectal adenomas were recruited via the "Identification and characterization of Inherited Predispositions to Colorectal Tumors" (UK CORGI) study. As part of the Oxford-Illumina WGS500 project, we performed whole-genome sequencing of constitutional DNA extracted from peripheral blood lymphocytes from 35 individuals with at least 10 colorectal adenomas before age 60 who tested negative for polyposis- and colorectal cancer predisposing genes in routine diagnostics.[2] The Illumina HiSeq platform was used and a median of ~40X coverage achieved. Read alignment and variant calling were performed using BWA, Stampy and Platypus as described.[2] Samples were additionally joint called using GATKv3 and annotated using ANNOVAR.[2] We extracted all variants predicted to result in protein truncation (nonsense, frameshift, or splice site variants) and prioritized for homozygous and compound heterozygous variants with a low frequency (MAF < 0.01) in the general population.

ii) Individuals (n = 74) with 10 or more colorectal adenomas with or without colorectal cancer who had been referred to specialist clinical genetics services for investigation of a possible inherited polyposis syndrome, but in whom genetic testing including *APC* and *MUTYH* had not revealed a genetic cause were recruited after giving informed consent to participate in the 'Genetic Mechanism in Polyposis of the Bowel' study

(REC 12/WA/0071). Constitutional DNA was extracted from peripheral blood lymphocytes. Whole-exome libraries were prepared using the TruSeq DNA Exome kit (Illumina) according to the manufactures instructions. Libraries were normalized, pooled and sequenced using a 75-base paired-end dual index read format on the Illumina HiSeq4000 according to the manufacturer's instructions. Read alignment and variant calling were performed using BWA against GRCh37h (1000 genomes version human_g1k_v37.fasta). BAM files were subjected to post-processing using samtools (sorted, fixmate, rmdup) to fix mate pairs and remove duplicates. Variants were called using GATK HaplotypeCaller on the recalibrated bam files. Variants were annotated using VEP, outputting all available annotation data. We extracted variants predicted to result in protein truncation (nonsense and frameshifting) and prioritized for homozygous and compound heterozygous variants with a low frequency (MAF <1%) in the general population.

iii) Colorectal cancer affected families with blood and tumor specimens available were selected for WGS and WES from the Australasian Colorectal Cancer Family Registry (ACCFR). Families with a pathogenic variant within a known hereditary CRC and polyposis susceptibility gene were excluded. Eighty-seven families comprised of 198 CRC- and early-onset polyp affected people were selected for sequencing based on having a family history of CRC that met one of the following criteria: 1) met the definition of Familial Colorectal Cancer Type X (FCCTX; *n* = 55), 2) Amsterdam II clinical criteria (AMII) with 2 of the defining triad being CRC-affected (n = 4), 3) >2 CRC-affected family members within the same blood line but not meeting FCCTX or AMII criteria (MCF; *n* = 28). Written informed consent was obtained from all study participants and the study protocol approved by Human Research Ethics Committees at the University of Melbourne (HREC#1954921). Germline whole exome sequencing (WES) was performed using SureSelect Human All Exon V4 (51Mb) kit (Agilent, Santa Clara, CA, USA) and 100bp paired-end sequencing on the Illumina HiSeq2000 to a mean coverage of 100x (Macrogen Inc., South Korea). Whole genome sequencing (WGS), using Illumina TruSeq DNA sample preparation and 100bp paired-end sequencing on the Illumina HiSeq2000 to a mean coverage of 30x (Macrogen Inc., South Korea). Sequence reads were mapped to the Human Reference Genome GRCh37 using BWA (v 0.7.12). Germline single nucleotide variants (SNVs) and short insertions and deletions (INDELs) were calculated using the GATK best practices pipeline (v 4.0.0) and we extracted variants predicted to result in protein truncation (nonsense and frameshifting) and prioritized for homozygous and compound heterozygous variants with a low frequency (MAF <1%) in the general population.

UK 100,000 genomes samples (100KGP) - We searched the genome sequencing data available (ISAAC pipeline) for 17,243 Caucasian participants of the rare diseases programme (v6 release, participants selected for phenotypes with no increased risk of cancer), 2,438 Caucasian CRC individuals included in the cancer programme (v8 release) and 283 Caucasian individuals with multiple bowel polyps (143 v6 rare diseases, 140 pilot project) for germline coding or splicing variants in *MBD4*. Variants were prioritized as described below.

UKBiobank samples - We searched the exome and genome sequencing data available for 200,000 participants in UKBiobank (exome sequencing; October 26th 2020 release) for germline coding or splicing variants in MBD4. This research was conducted under UK Biobank application number 8508. Variants were prioritized as described below.

GnomAD - We searched the full and non-Finnish European populations of the gnomAD database (v2.1 dataset) for germline coding or splice site variants in *MBD4*. Variants were prioritized as described below.

Molecular inversion probe sequencing of *MBD4* - All participants in the MIP screen provided written informed consent. Details of the inclusion criteria are described in **Table S1**. This study was approved by the local medical ethics committee (CMO light; study number 2015/2172 of the Radboudumc Nijmegen). Leukocyte-derived DNA was used for targeted resequencing of *MBD4* (NM_003925.2) using 32 Molecular Inversion Probes (MIPs), covering all coding regions and intron-exon boundaries, were designed according to the previously published methodology.[3,4] After targeted capture, samples were sequenced on a NextSeq500 (Illumina) system. Reads were mapped using BWA and variants called using GATK's UnifiedGenotyper. After variant calling, all variants with an at least 40-fold absolute coverage, ≥20 variant reads, ≥25% variant reads and ≥8.000 quality by depth scores were selected for further analyses. Loss of function (LOF) variants in *MBD4* (see below) identified using MIP-sequencing with a quality by depth score of 8.000-11.000 were validated using Sanger sequencing.

Sanger sequencing of *MBD4* - Bidirectional Sanger sequencing of the *MBD4* open reading frame and flanking intronic regions was undertaken in blood DNA samples from 118 individuals from the UK 'Genetic Mechanisms in Polyposis of the Bowel' study who were not included in the WES screening for germline pathogenic variant. All had at least 10 colorectal adenomas and had tested negative for pathogenic variants in *APC* and *MUTYH* in an NHS diagnostic setting. Primer design (available upon request) was carried out using Primer3 and primers checked for template (NG_033106.1) specificity using Primer BLAST. PCR was carried out using MegaMix Gold (Microzone). PCR products were clean-upup using exonuclease (New

England Biolab) and shrimp alkaline phosphatase (ThermoFisher) and subjected to Sanger sequencing on an ABI 3730 analyser (Applied Biosystems). Sanger chromatograms were visualised with Sequencher (Gene Codes, USA).

MBD4 variant interpretation - All *MBD4* variants located in an exon region, canonical splice site (positions +1, +2 and −2, -1), and coding or noncoding splice site region (3' splice site -12 till +2 and 5' splice site -3 till +6) were included for further analyses. Furthermore, only variants with an allele frequency <1% in an in-house database of 12,244 germline exomes that have been sequenced at Genome Diagnostics Nijmegen (https://order.radboudumc.nl/en/genetics) and <2% in ExAC and gnomAD were included. To select variants of pathogenic potential, we selected all frameshift and nonsense variants, and missense variants with a PhyloP score ≥3 and a CADD_PHRED score ≥15. For variants with a predicted splicing effect of more than 20% by SpliceAI additional in silico splice site predictions were obtained using MaxEntScan, NNSPLICE, and Human Splicing Finder (Alamut Visual 2.13). Splice site losses were included when 1) the variant splice score was less than 50% of the scoring range for at least two algorithms and 2) the difference between wildtype and variant splice score was more than 20% of the scoring range in at least three algorithms. Splice site gains were included when 1) the variant splice score was above 75% of the scoring range for at least two algorithms and 2) the difference between the gained splice site and the nearest splice site was more than 2% of the scoring range in at least two algorithms.

Whole-exome sequencing of adenomas - WEHI-2 (previously reported as WEHI-AML-2) consented to the use of their clinical information and tissues for research in accordance with the Declaration of Helsinki. The project was approved by human research ethics committees from the Walter & Eliza Hall Institute of Medical Research (WEHI) and Melbourne Health (MH) (WEHI HREC project 13/01, MH HREC project 2012.274). DNA was extracted from nine fresh frozen adenomas, two formalin fixed paraffin embedded (FFPE) adenomas and three fresh frozen macroscopically normal bowel tissues from D:II-1, nine independent FFPE adenomas from WEHI-2 **(Figure S1H-I; Table S2)**. Exome library preparations were performed according to the manufacturer using either the i) Agilent SureSelectXT Human All Exon V6 (Agilent Technologies), ii) Agilent SureSelect XT Low Input Human Whole Exome V6 (Agilent Technologies) or iii) Illumina TruSeq exome (Illumina) enrichment kit in combination with sequencing on a NextSeq500 (Illumina) or NovaSeq 6000 (Illumina) **(Table S2)**.

WES sequencing reads from D:II-1 were aligned with BWA and stampy v1.0.28. Duplicates were marked using picard 1.9.2. Clonal tracking was performed with default superFreq (v1.3.2)[5] using preliminary variants from VarScan (v2.3)[6] with the options --strand-filter 0, --p-value 0.05 and --min-var-freq 0.02. For the FFPE samples >700,000 variants were

called per sample using these settings compared to ~100,000 in the fresh frozen samples. Varscan VCFs from FFPE samples were additionally filtered to remove variants with <10% VAF and <4 reads supporting the variant allele. Somatic variants were called using Mutect2 (GATK version 4.1.0.0). Normal bowel samples from the caecum and transverse colon of simplex case D:II-1 were used as matched normal, a panel of 6 normal colon samples from other persons without CRC or polyps were used as a panel of normals reference to assist with filtering platform artefacts. “af-only-gnomad.raw.sites.b37.vcf” was provided as an additional germline reference.

WES sequencing reads from WEHI-2 were aligned to hg19 with BWA[7] Variant calling, CNA calling and clonal tracking was done with default superFreq (v1.3.2)[5] using preliminary variants from VarScan (v2.3)[6] with the options --strand-filter 0, --p-value 0.01 and --min-var-freq 0.05. To limit artefacts, the variants were further filtered based on clonal assignment.

For each adenoma, high confidence somatic mutations were identified as described previously with minor modifications.[8] In brief, somatic variants covered by ≥15x sequencing reads, ≥10% variant allele frequency, and ≥6 variant reads, and (for variants called by Mutect2) with ≥2 variant reads per read pair (to exclude FFPE artefacts) were included. WEHI-2 received an allogeneic bone marrow transplant and variants contributed by the haematopoietic stem cell donor were removed based on clonal tracking in SuperFreq.[5] For somatic variants identified by SuperFreq, a mean read depth >30 across all samples was required and excluded variants if they were detected above 3% VAF (supported by at least 2 reads) in a sample where the clone was deemed absent (clonality <1%). A representative set of somatic mutations were validated by Sanger sequencing or by processing micro-dissected material from the adenomas with the TruSight Tumor 26 Kit (Illumina), including one additional adenoma from WEH-2 (see also **Table S2**).

WES methods for the sporadic adenoma samples was previously described.[9] Somatic variants were called from the BAMs for each region, which were previously aligned to hg19, using Mutect2, following the same strategy as described for D:II-1. Each adenoma sample had a matched normal. In all analyses the union of mutations called in any of the regions from the same adenoma were combined.

Somatic mutation spectrum and driver gene analyses - The number of somatic mutations per megabase (Mb), mutation spectrum and the number of CpG>TpG transitions was determined. A linear model describing the number of CpG>TpG transitions as a function of age was analyzed in base R. Methylation status of the sites with somatic mutation was assessed in public whole genome bisulfite sequencing (WGBS) data from normal sigmoid colon from the Roadmap Epigenomics Consortium.[10] The contribution of mutational signatures to the somatic

mutation spectrum was inferred using the R package MutationalPatterns[11] in combination with COSMIC-v3 mutational signatures.

To compare to the The Cancer Genome Atlas (TCGA) CRC data[12], we downloaded variant calls from SomaticSniper, VarScan2, MuTect2, and MuSE through the National Cancer Institute Genomic Data Commons. Variants were retained if the variant allele frequency was greater than 20% with at least 20 read depth, and if it was identified by at least 3 of the 4 callers. Mismatch repair status was available for a subset of samples, which we used to classify tumors as microsatellite stable (MSS) or unstable (MSI). All non-synonymous somatic variants in cancer driver genes reported by The Cancer Gene Census (CGC v92) in COSMIC were extracted for each of the sequenced adenomas. Next, driver genes were prioritised with their previous associations as a colorectal cancer driver by TCGA and Dietlein et al.[12,13] To compare driver genes and mutation types, genes that were mutated significantly different in the MBD4-deficient individuals or sporadic adenomas were plotted in an oncoprint.

CRISPR/Cas9 generated *MBD4* knockout cells - HAP1 cells were maintained in Iscove's Modified Dulbecco's Medium (IMDM; GIBCO), containing 10% fetal calf serum (FCS), 1% glutamine, and 1% penicillin/streptomycin. Single guide RNAs (sgRNA) targeting the glycosylase domain of *MBD4* were designed using CHOPCHOP, cloned into the Cas9 expression vector PX459 v2.0 (Addgene plasmid #62988) and HAP1 cells were transfected according to the method described by Ran *et al.*[14] with minor modifications. After puromycin selection single cell clones were derived using a FACS sorting. Effective knockout of *MBD4* was determined based on Sanger sequencing of the target regions, mRNA expression and by Western blot. Full *MBD4* knockout (*MBD4*^{KO}) HAP1 single cell clones were cultured for 142 days, followed by another single cell sort. Subclones were expanded for 14 days and gDNA was isolated from the parental HAP1 clone and *MBD4*^{KO} subclones. Two WT and *MBD4*^{KO} clones were whole-genome sequenced using the TruSeq DNA PCR-Free library kit (Illumina) and sequenced on a NovaSeq 6000 System (Illumina). Reads were mapped using BWA and for all samples at least 90% of the genome was covered at 20X. Variant calling and mutational signature analysis was performed as described above. As modification to the analysis the average 96-profile of the HAP1 WT clones was extracted from the *MBD4*^{KO} clone to be left with the 96-profile specific to the *MBD4* knockout.

Assessment of mutation rate in a mouse model of MBD4 deficiency - Whole genome sequencing was performed on individual mouse haematopoietic progenitor colonies as previously described.[1] In brief, mouse bone marrow cells were cultured in semi solid agar. Each culture contained 10,000 bone marrow cells, suspended in Dulbecco modified Eagle medium with 20% bovine calf serum and 0.3% agar, with 100ng murine stem cell factor, 10ng

murine IL-3 and 2 IU erythropoietin. Cells were incubated for 11 days at 37°C in a humidified atmosphere with 10% CO_2. DNA was extracted from individual colonies using QIAamp DNA Micro Kit (Qiagen), amplified using TruePrime WGA Kit (SYGNIS) and purified using QIAamp DNA Mini Kit (Qiagen). Whole genome sequencing was performed on the NovaSeq 6000 (150bp paired end reads, Illumina). The mouse sequencing data was aligned to the mouse genome (mm10) using bwa-mem. WGS was also performed on the original bone marrow DNA and used to identify variants unique to the individual colony. Results from the wildtype and knockout colonies were reported previously and were deposited at SRA (Accession: PRJNA419992).[1]

Generation of a lymphoblastoid cell line from simplex case D:II-1 - Peripheral blood lymphocytes (PBLs) were isolated using Ficoll-Paque PLUS (eppendorf) following manufacturers instructions from a fresh blood sample, collected in sodium heparin tubes from simplex case D:II-1. A lymphoblastoid line was generated by Epstein Barr virus transformation by the Culture collections team, Public Health England.

RNA and protein analysis - Taqman expression probes HS01023548 and HS00187498 were used to quantify *MBD4 m*RNA extracted from a lymphoblastoid cell line from simplex case D:II-1 (further details available upon request). Protein lysates from cells were analysed by western blotting using anti-MBD4 antibody ab224809 (Abcam). HCT116, HAP1 and D:II-1 lymphoblastoid cells were resuspended in RIPA buffer (Thermo Fisher Scientific). Total lysates were quantified with Pierce BCA Protein Assay kit (Thermo Fisher Scientific) according to manufacturer's instructions. 20 μg of protein lysate were loaded on a 4-20% gradient gel (Thermo Fisher Scientific) or NuPage 4-12% Bis-Tris Gels (Invitrogen). After transfer with iBlot2 dry Blotting System (Thermo Fisher Scientific) and blocking membranes were blotted for anti-MBD4 (abcam, diluted 1:1000) and anti-α-tubulin (Sigma, 1 diluted 1:5000 or Abcam, diluted 1:500) as a loading control. Membranes were exposed to hyperfilm ECL (GE Healthcare) and developed using a X-Ray Film Processor (Konica) or scanned on the Odyssey Infrared Imaging System (Li-COR).

MBD4 immunohistochemistry - Formalin-fixed paraffin-embedded (FFPE) specimens of colorectal adenomas were analysed by immunohistochemistry using the same anti-MBD4 antibody. Samples sections (5 μm) were deparaffinized with xylene and rehydrated. Sections were incubated with 6% H_2O_2 for 20 min at room temperature to block endogenous peroxidase activity. Antigen retrieval was carried out by incubating the slides in citrate buffer (pH 6.0) at 95°C for 10 min. Sections were blocked with goat serum for 30 min at room temperature. Primary antibodies were diluted in 1% goat serum/0.1% BSA/PBS.

Sections were incubated with MBD4 primary antibody (ab224809, 1:50) overnight at 4°C. Sections were washed with Tris-buffered saline with 0.1% Tween 20 (TBST) and incubated with secondary antibody anti rabbit (PK-6100) for 30 min. Tertiary (ABC Biotinylated) was kept for 30 min. Staining was visualized using a HRP/DAB detection system Dako. Control IHC experiments (data not shown) were performed without primary antibody. All sections were counterstained with Gill's haematoxylin and mounted for digital slide scanning using a Zeiss ActioScan Z1.

Acknowledgements: We thank all families for their collaboration. We thank Dr. Robbert D.A. Weren, Dr. Janet Vos and Eveline Kamping for technical assistance, the Genome Technology Platform for MIP sequencing support, Dr. Christian Gillissen for the use of the annotation pipeline in the Radboudumc, Nijmegen, and Dr. Peggy Manders for the use of samples from the Dutch Parelsnoer Institute Biobank Hereditary Colorectal Cancer. We thank expert colonoscopists in Cardiff, Drs Gareth Thomas and Sunil Dolwani. We acknowledge expert technical assistance from the Austin Molecular Laboratory and the Australian Genome Research Facility for providing expert technical assistance with genomic analysis of cancer samples. We thank the Wales Gene Park for expert help with WES and processing of data, Loys Richards and Laura Butlin for their expert assistance with research governance and Karen Bailey for help with study participant recruitment. We thank members of the Colorectal Oncogenomics Group and the participants and staff from the Colon-CFR in particular, Maggie Angelakos, Samantha Fox and Allyson Templeton for their support of this study. Computation and bioinformatics were also provided by Melbourne Bioinformatics on its Peak Computing Facility. Furthermore, we thank the Birmingham Genomics Service at The University of Birmingham, for the generation of the sequencing and methylation array data. Some of the computations described in this paper were performed using the University of Birmingham's BlueBEAR HPC service, which provides a High Performance Computing service to the University's research community. We thank all participants of the CORGI study for their collaboration. This research was made possible through access to the data and findings generated by the 100,000 Genomes Project. The 100,000 Genomes Project is managed by Genomics England Limited (a wholly owned company of the Department of Health and Social Care). The 100,000 Genomes Project is funded by the National Institute for Health Research and NHS England. The Wellcome Trust, Cancer Research UK and the Medical Research Council have also funded research infrastructure. The 100,000 Genomes Project uses data provided by study participants and collected by the National Health Service as part of their care and support.

REFERENCES

1. Sanders MA, Chew E, Flensburg C, et al. MBD4 guards against methylation damage and germ line deficiency predisposes to clonal hematopoiesis and early-onset AML. Blood 2018;132:1526-1534.
2. Palles C, Cazier JB, Howarth KM, et al. Germline mutations affecting the proofreading domains of POLE and POLD1 predispose to colorectal adenomas and carcinomas. Nat Genet 2013;45:136-44.
3. O'Roak BJ, Vives L, Fu W, et al. Multiplex targeted sequencing identifies recurrently mutated genes in autism spectrum disorders. Science 2012;338:1619-22.
4. Boyle EA, O'Roak BJ, Martin BK, et al. MIPgen: optimized modeling and design of molecular inversion probes for targeted resequencing. Bioinformatics 2014;30:2670-2.
5. Flensburg C, Sargeant T, Oshlack A, et al. SuperFreq: Integrated mutation detection and clonal tracking in cancer. PLoS Comput Biol 2020;16:e1007603.
6. Li H, Durbin R. Fast and accurate short read alignment with Burrows-Wheeler transform. Bioinformatics 2009;25:1754-60.
7. Koboldt DC, Zhang Q, Larson DE, et al. VarScan 2: somatic mutation and copy number alteration discovery in cancer by exome sequencing. Genome Res 2012;22:568-76.

8. Grolleman JE, de Voer RM, Elsayed FA, et al. Mutational Signature Analysis Reveals NTHL1 Deficiency to Cause a Multi-tumor Phenotype. Cancer Cell 2019;35:256-266
9. Cross W, Kovac M, Mustonen V, et al. The evolutionary landscape of colorectal tumorigenesis. Nature ecology & evolution 2018;2:1661-1672.
10. Roadmap Epigenomics Consortium; Kundaje A, Meuleman W, Ernst J, et al. Integrative analysis of 111 reference human epigenomes. Nature 2015;518:317-330.
11. Blokzijl F, Janssen R, van Boxtel R, et al. MutationalPatterns: comprehensive genome-wide analysis of mutational processes. Genome Med 2018;10:33.
12. Cancer Genome Atlas Network. Comprehensive molecular characterization of human colon and rectal cancer. Nature 2012;487:330-337.
13. Dietlein F, Weghorn D, Taylor-Weiner A, et al. Identification of cancer driver genes based on nucleotide context. Nat Genet 2020;52:208-218.
14. Ran FA, Hsu PD, Wright J, Agarwala V, Scott DA, Zhang F. Genome engineering using the CRISPR-Cas9 system. Nat Protoc. 2013;8(11):2281-2308.
